# 8 specific Chinese herbal injections combined with chemotherapy for breast cancer: a systematic review and network meta-analysis of comparative safety and efficacy

**DOI:** 10.3389/fphar.2025.1661803

**Published:** 2025-10-03

**Authors:** Yunze Shi, Jianhao Cheng, Yichen Zhao, Long He, Lifeng Duan, Huichao Wang, Xiaoyang Hu

**Affiliations:** ^1^ Department of Internal Medicine of Chinese Medicine, School of Graduate, Heilongjiang University of Chinese Medicine, Harbin, Heilongjiang, China; ^2^ Department of Chinese Formulae, Heilongjiang University of Chinese Medicine, Harbin, China

**Keywords:** Chinese herbal injection, breast cancer, chemotherapy, network meta-analysis, systematic evaluation

## Abstract

**Background:**

Breast cancer (BC) poses a major threat to human health. Since the beginning of the 21st century, numerous clinical studies conducted in China have reported the therapeutic advantages of combining Chinese herbal injections (CHIs) with chemotherapy; however, comparative evaluations of different CHIs remain scarce. This multidimensional network meta-analysis was designed to compare the efficacy of various CHIs and to identify the optimal combination regimen of CHI plus chemotherapy for breast cancer treatment.

**Methods:**

By searching multiple databases, we screened randomized controlled trials (RCTs) on Chinese herbal injections (CHIs) combined with chemotherapy interventions for breast cancer (BC) from database inception to 25 October 2024. Studies meeting inclusion criteria with methodologically sound quality were included. Data analysis was performed using Stata 17.0 and R 4.2.1 software, with odds ratio (OR) and standardized mean difference (SMD) as effect size measures. The surface under the cumulative ranking curve (SUCRA) method was applied to rank the evaluated treatment regimens.

**Results:**

This network meta-analysis included 44 eligible RCTs, involving 3,982 patients and 8 CHIs. Shenqifuzheng Injection (SQFZ) combined with chemotherapy was most effective in enhancing the efficacy of chemotherapy, improving the quality of life of breast cancer patients, ameliorating the CD4+/CD8+ T-cell ratio, and inhibiting the decrease in white blood cell (WBC) count after chemotherapy. Compound Sophora Injection (FFKS) combined with chemotherapy performed best in increasing post - chemotherapy CD4^+^ and CD8^+^, lowering tumor marker CA125, and reducing post-chemotherapy platelet (PLT) and hemoglobin (HGB) declines. Kangai Injection (KA) combined with chemotherapy was most effective for CD3^+^ improvement. Kanglaite Injection (KLT) combined with chemotherapy had the best effect on reducing tumor markers CEA and CA153. Although a 2021 network meta-analysis (Comparative Efficacy and Safety of Chinese Herbal Injections Combined With Cyclophosphamide and 5-Fluorouracil Chemotherapies in Treatment of Breast Cancer: A Bayesian Network Meta-Analysis) examined chemotherapy combined with Chinese medicine injections for breast cancer, it was limited to the CF regimen and assessed few outcomes, with some lower-quality studies included (excluded herein). Our study improves methodology by incorporating high-quality RCTs across all chemotherapy regimens and evaluating multiple outcomes. This provides more comprehensive results, identifying SQFZ as most effective for improving response rate and Karnofsky Performance Status (KPS), thereby enhancing clinical utility.

**Systematic Review Registration:**

https://www.crd.york.ac.uk/PROSPERO/myprospero, identifier [CRD42024589306].

## 1 Introduction

Breast cancer (BC), a malignant tumor originating in the epithelial tissue of the breast, ranks among the most prevalent malignancies in women globally and is a leading cause of cancer-related mortality (Brahim et al., 2022; Sachs et al., 2018; Zeng et al., 2020). According to the latest data from the International Agency for Research on Cancer (IARC) under the World Health Organization, there were 19.96 million new cancer cases and 9.74 million cancer-related deaths globally in 2022. Notably, female breast cancer ranked as the second most commonly diagnosed cancer, with approximately 2.30 million new cases, accounting for 11.5% of all cancer cases (Hirko et al., 2022; Zhang et al., 2024). Current standard treatments for BC include radiotherapy, chemotherapy, surgery, hormonal therapy, endocrine therapy, and targeted therapy (Osaki et al., 2019). While chemotherapy remains a cornerstone in comprehensive management by inhibiting tumor cell proliferation and metastasis, it is often accompanied by severe toxic side effects, such as fatigue, insomnia, peripheral neuropathy, cognitive impairment, estrogen deprivation, and cardiotoxicity (Millon et al., 1965; Weymann et al., 2014). Enhancing therapeutic efficacy while minimizing these adverse effects remains an urgent clinical challenge (Akula et al., 2020).

Chinese Herbal injections (CHIs) (For detailed information on Chinese Herbal injections, please refer to Table 1), products integrating traditional Chinese medicine theory with modern pharmaceutical technology, have gained increasing attention in breast cancer treatment (Di Nardo et al., 2022; Huang and Wei, 2019) Characterized by their multi-component, multi-target, and multi-pathway mechanisms (Li et al., 2023), CHIs exhibit potential to modulate systemic immune function, enhance anti-cancer capacity, and mitigate chemotherapy-induced toxicity (Yang et al., 2023). Numerous studies have demonstrated the synergistic and detoxifying effects of CHIs combined with chemotherapy in cancer treatment, leading to their widespread clinical application (Yue et al., 2017). However, conventional meta-analyses focusing on single types of CHIs lack the capacity to horizontally compare and rank the efficacy of diverse CHIs (Li et al., 2020). Bayesian network meta-analysis (NMA) surpasses traditional meta-analytical methods by enabling simultaneous comparisons of multiple therapies, offering greater flexibility in modeling complex interventions, and generating scientifically robust interpretations of causal relationships (Yu et al., 2021; Zhang et al., 2015). This approach effectively handles intricate data structures and provides comprehensive evidence synthesis to identify optimal therapeutic outcomes. This study employs Bayesian NMA to evaluate the efficacy and safety of CHIs combined with chemotherapy for breast cancer, aiming to compare clinical outcomes across various injections. The findings are anticipated to guide rational drug selection in clinical practice, ultimately improving therapeutic efficacy and reducing adverse effects in breast cancer patients undergoing chemotherapy.

**TABLE 1:** Characteristics and composition of the eight Chinese herbal injections included in the network meta-analysis.

Abbreviation	Full title	Constituent herbs and authors	Family	Medicinal part	Source	Marker compounds	Extraction solvent and process
SQFZ	Shenqifuzheng Injection	Astragalus mongholicus [Bunge ]	Fabaceae	dried root	Kew Science	Astragaloside IV ≥ 0.08 mg/mL	6 times the volume of 70% ethanol, reflux extraction 3 times, 90 min each
		Codonopsis pilosula [Franch ]	Campanulaceae	dried root	Kew Science		
FFKS	Compound Sophora Injection	Sophora flavescens [Aiton ]	Fabaceae	dried root	Kew Science	Matrine and Oxymatrine ≥ 1 mg/mL	Double ethanol precipitation: 1st to 60% ethanol, refrigerated sedimentation 36 h; 2nd to 80%–90% ethanol, refrigerated sedimentation 6 h
		Smilax glabra [Roxb]	Smilacaceae	dried root	Kew Science	Astilbin ≥ 0.45 mg/mL	
AD	Aidi Injection	Astragalus mongholicus [Bunge ]	Fabaceae	dried root	Kew Science	Astragaloside IV ≥ 0.08 mg/mL	
		Eleutherococcus senticosus [Rupr. and Maxim ]	Araliaceae	dried root and rhizome or stem	Kew Science	Contains Syringin ≥ 0.05 mg/mL	
		Panax ginseng [C.A.Mey]	Araliaceae	dried root	Kew Science	Ginsenoside Rg1 and Re ≥ 0.27 mg/mL, ginsenoside Rb1 ≥ 0.18 mg/mL	
		Cantharis [Linnaeus]	Cantharidae		Global Biodiversity Information Facility	Cantharidin ≥ 0.35 mg/mL	
HQ	Huangqi Injection	Astragalus mongholicus [Bunge ]	Fabaceae	dried root	Kew Science	Astragaloside IV ≥ 0.08 mg/mL	queous extract; Residue → ethanol reflux → ethanolic extract; Combine → high-conc. Ethanol precipitation (83%–87%)
KA	Kangai Injection	Panax ginseng [C.A.Mey]	Araliaceae	dried root	Kew Science	Ginsenoside Rg1 and Re ≥ 0.27 mg/mL, Ginsenoside Rb1 ≥ 0.18 mg/mL	Extraction and Concentration: Ginseng and Astragalus extracts are added directly. Sophora flavescens is extracted and then incorporated. Concentration can be performed either during extraction or after combining extracts Purification: The process includes water precipitation, activated carbon adsorption (optimal time: 48 h), and a two-step ultrafiltration (100KD followed by 10KD membranes) to remove macromolecular impurities, allergens, and pyrogens Sterilization: Final product is sterilized with no significant impact on active content or allergenic potential
		Astragalus mongholicus [Bunge ]	Fabaceae	dried root	Kew Science	Astragaloside IV ≥ 0.08 mg/mL	
KLT	Kanglaite Injection	Coix lacryma-jobi [L]	Poaceae	Seed	Kew Science	Trilaurin ≥ 0.5 mg/mL	
HCS	Cinobu facini Injection	*Bufo gargarizans* [Cantor]	Bufonidae	toad skin	Global Biodiversity Information Facility		
SM	Shenmai Injection	Panax ginseng [C.A.Mey]	Araliaceae	dried root	Kew Science	Ginsenoside Rg1 and Re ≥ 0.27 mg/mL, ginsenoside Rb1 ≥ 0.18 mg/mL	Ginseng: 75% EtOH reflux (4 × 2 h); Ophiopogon: water decoction (3 × 1 h); Final prep: combined extracts, 0.2% activated carbon, pH = 8.0, 0.6% polysorbate 80, autoclave 121 °C/8min
		Ophiopogon intermedius [D.Don]	Asparagaceae	Not specified	Kew Science	Total ophiopogon saponins, expressed as ruscone, ≥0.12 mg/mL	

## 2 Characterization of interventions: chinese herbal injections (CHIs)

To ensure taxonomic precision and pharmacological reproducibility, all Chinese Herbal Injections (CHIs) evaluated in this network meta-analysis are clearly defined in Table 1. The botanical identities of the constituent species were verified using Kew Science (http://mpns.kew.org/mpns-portal), and the identities of certain medicinal animals were cross-referenced with the GBIF database (https://www.gbif.org/). The table provides a detailed listing of the herbal components of each injection—including full title, Constituent Herbs, Family, medicinal parts, and naming authorities—as well as key marker compounds for quality control and a brief description of the extraction process. SQFZ is composed of Codonopsis and Astragalus (Gardner et al., 2000), the quantitative markers and extraction process are sourced from (Huang and Zhang, 2009). FFKS is composed of the natural medicines Sophora flavescens and Smilax glabra (Zhou et al., 2025). Its process and markers are detailed in the table, sourced from (Liu, 2011; Liu et al., 2011). Aidi is primarily composed of extracts from Astragalus, Eleutherococcus senticosus, Ginseng, and Cantharis (Burn, 1967). The markers are sourced from (Chen et al., 2011). Kangai is primarily made from Ginseng and Astragalus (Zhu and Tian, 2024). The process and quantitative markers are sourced from (Bi et al., 2024; Tang, 2018). The main component of Kanglaite Injection is coix seed. (Liu et al., 2020). Quantitative marker information is sourced from (Wang et al., 2021). HCS’s main component is toad skin (Meng et al., 2009). SM’s main components are Ginseng and Ophiopogon. The quantitative markers and extraction process are sourced from (Wu et al., 2005). Due to the proprietary manufacturing techniques maintained by the producers, some details in the table are not available.

## 3 Methods

The meta-analysis was conducted in strict accordance with the Preferred Reporting Items for Systematic Reviews and Meta-Analyses (PRISMA) guidelines and their specific requirements for Network Meta-Analysis (NMA). The study protocol has been registered in an International Prospective Register of Systematic Reviews (PROSPERO), with the registration number CRD42024589306.

### 3.1 Search strategy

We conducted a comprehensive literature search across eight electronic databases (Web of Science, Embase, PubMed, Cochrane Library, CNKI, Wanfang, SinoMed and VIP) from inception to 25 October 2024. The search strategy combined Medical Subject Headings (MeSH) and free-text terms covering three concept groups:

1. Breast cancer–MeSH: “Breast Neoplasms”, etc.; free-text: Breast*Cancer, 乳腺肿瘤(Breast Tumor), 乳癌(breast cancer), mammary malignancy, etc. (full list in Supplementary File 1).
2. Chinese herbal injections–MeSH: “Medicine, Chinese Traditional, etc.”; free-text: Chinese herbal injection, 参芪(Shenqi), Shenqi, Kangai, 华蟾素 (Cinobu facini Injection), etc. (encompassing all evaluated injections: SQFZ, FFKS, KA, KLT, …).
3. Study design–free-text: random*, 随机 (Random) (to identify RCTs). Boolean operators structured all queries. Reference lists of eligible studies and clinical-trial registries (ClinicalTrials.gov, WHO ICTRP) were hand-searched to supplement electronic retrieval. After deduplication in EndNote 20, 9,796 records were screened. Complete search syntax for each database is provided in Supplementary File 2.

### 3.2 Inclusion and exclusion criteria

According to the PICOTS principle, studies meeting the following criteria were included:

1. Study population: Patients included were diagnosed with breast cancer (BC) via cytological or pathological methods, with TNM stages 1–4, and of the female sex, regardless of age, race, region, or nationality. Patients with other concurrent tumors were excluded.
2. Interventions: The study group received a combination of Chinese herbal injection (CHI),The chemotherapy regimens were not fixed.
3. Control measures: The control group received chemotherapy alone,The chemotherapy regimens were consistent with the intervention groups.
4. Outcome measures: Studies evaluated the following outcomes:
  a. Efficacy: Assessed according to the World Health Organization (WHO) criteria for solid tumor efficacy. Responses were categorized. Complete Response (CR): Complete disappearance of all visible lesions within 1 month after treatment. Partial Response (PR): Reduction of ≥50% in the size of a single tumor or >50% reduction in the product of the perpendicular diameters of the two largest tumors in multiple lesions. Stable Disease (SD): No significant change in the patient’s condition for at least 4 weeks, with an estimated reduction in tumor size of <25% or an increase of <50%. Progressive Disease (PD): An increase of ≥25% in the estimated size of new or existing lesions. In this study, complete response (CR) and partial response (PR) were considered effective, while stable disease (SD) and progressive disease (PD) were considered ineffective.
  b. Quality of life: Assessed using the Karnofsky Performance Status (KPS) scale. An increase of ≥10 points post-treatment compared to pre-treatment was considered an improvement, a decrease of ≥10 points indicated a decline, and changes of <10 points were classified as stable. In this study, patients with improved quality of life were considered effective, while those with stable or declined conditions were considered ineffective. The numbers of effective and ineffective cases were statistically analyzed.
  c. Immune cell indicators: Included CD3^+^, CD4^+^, CD8^+^, and CD4+/CD8+ ratios.
  d. Tumor markers (biochemical substances produced by tumor cells or host responses): Included CA125, CA153, and CEA.
  e. Adverse reactions: Included white blood cell count (WBC), platelet count (PLT), and hemoglobin (HGB).
5. Time: Assessment was conducted at the end of the chemotherapy cycles.
6. Study type: Only randomized controlled trials (RCTs) were included.

Studies were excluded if they met any of the following criteria: 1. Animal or cell experiments, case reports, research proposals, review articles, letters, editorials, or conference abstracts; 2. Missing data or significant errors; 3. Duplicate publications; 4. Unavailable full texts.

Transitivity Assessment Method:Transitivity was evaluated across four key domains: 1 Baseline similarity of participants (e.g., age, TNM stage); 2 Methodological homogeneity of trials (e.g., design, blinding); 3 Network structure (star-shaped network where all interventions were compared with chemotherapy alone); 4 Clinical homogeneity of interventions (all CHIs were adjunctive to chemotherapy).

Consistency Assessment Method: The methods for consistency assessment should be determined based on the network structure (i.e., whether closed loops exist). Node-splitting can be used to test local inconsistency, and ultimately, global inconsistency can be evaluated by comparing the Deviance Information Criterion (DIC) between the consistency and inconsistency models.

### 3.3 Study selection process

**FIGURE 1:**
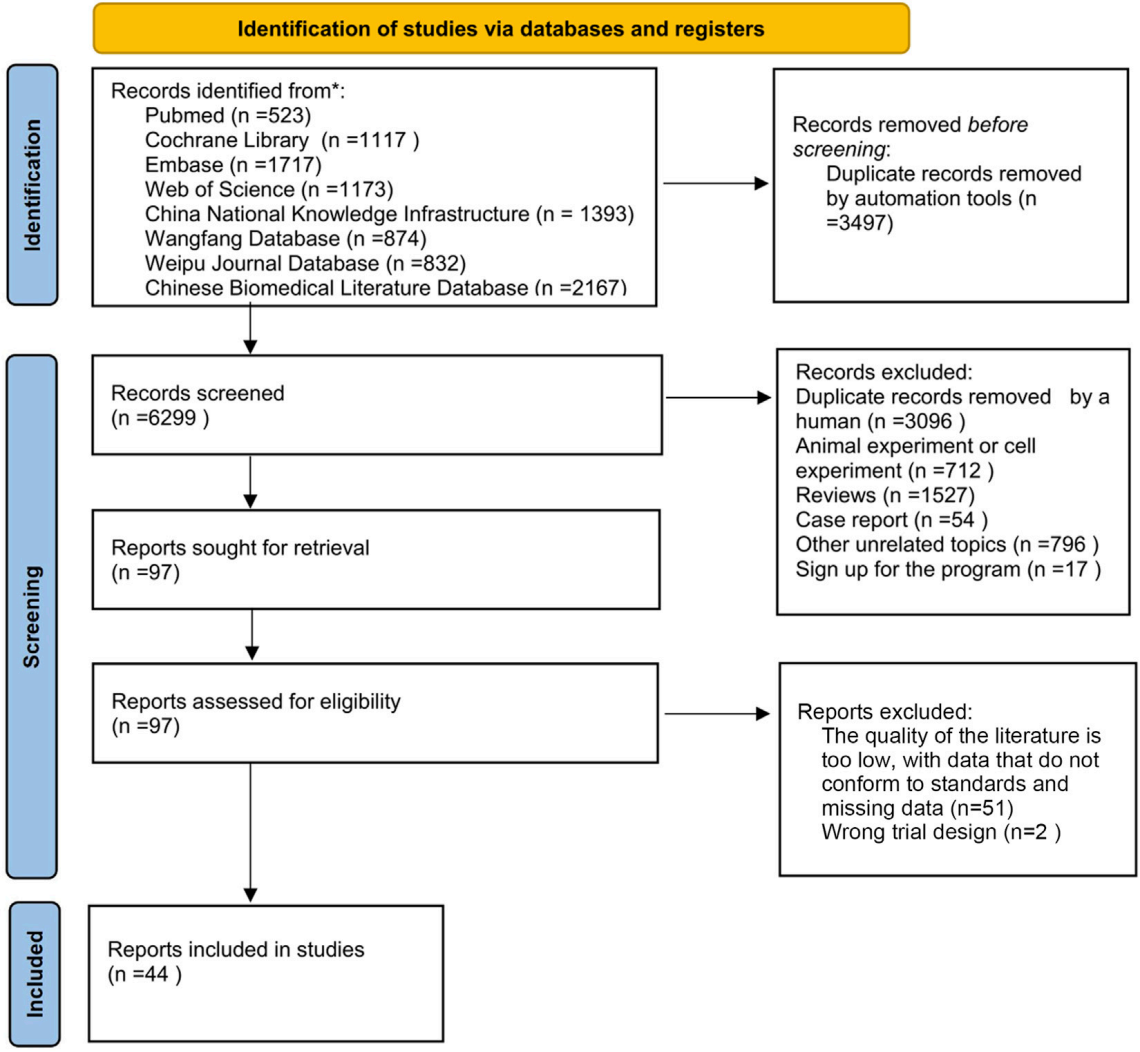
Flowchart.

The selection of studies followed a systematic and predefined process in accordance with PRISMA guidelines (Page et al., 2021). After comprehensive database searches and removal of duplicates, the initial pool of 9,796 records underwent a two-step screening procedure: Title and Abstract Screening: Two researchers (YS and JC) independently screened the titles and abstracts of all retrieved records against the predefined inclusion and exclusion criteria (Section 3.2). Studies clearly irrelevant to the research question were excluded at this stage. Any disagreements were resolved through discussion or consultation with a third researcher (YH). Full-Text Assessment: The full texts of potentially relevant articles identified in the first step were obtained. The same two researchers (YS and JC) independently evaluated these full texts against the inclusion/exclusion criteria. Studies failing to meet any criterion were excluded. Reasons for exclusion at the full-text stage were recorded. Disagreements were again resolved through discussion or third-party consultation (YH). The brief process of screening and inclusion is also depicted in Figure 1 (PRISMA Flow Diagram).

### 3.4 Data extraction

Two researchers independently extracted data using Excel 2016, recording: first author, publication year, country, randomization/blinding details, intervention/control group descriptions, treatment duration, population characteristics, and outcome measures. To ensure accuracy, a reviewer agreement protocol was implemented:

1. Title/abstract screening: Inter-reviewer reliability (Kappa ≥ 0.85) was confirmed via pilot testing
2. Full-text screening: Disagreements (<5%; 2/44 studies) were resolved through discussion or third-reviewer arbitration
3. Data extraction: Cross-verification achieved 98% consistency for numerical variables and 100% for categorical variables

Extracted data were compiled into a baseline characteristics table (Table 2).

**TABLE 2:** Baseline characteristics of the included studies.

Study	Samplesize (T/C)	Age (T/C)	Gender	TNM stage	Intervention	Treatment	Control	Outcomes	Control type	Blinding	Multicenter
A2011	80 (40/40)	28–65	Female	Ⅲ,Ⅳ	SQFZ	(A): SQFZ (250 mL qd 21d × 3 cycles) + (B)	(B): TPX (175 mg 21d × 3 cycles) + EADM (70 mg 21d × 3 cycles)	①	Chemotherapy alone	Not reported	**No**
Shen2016	68 (34/34)	29–71/31–72	Female	Ⅱ,Ⅲ	FFKS	(A): FFKS (20 mL + 0.9% 250 mL NS qd 21d × 6 cycles) + (B)	(B)Docetaxel (75 mg/m^2^ qd 21d × 6 cycles) + Epimedium (50 mg/m^2^ qd 21d × 6 cycles) + Cyclophosphamide (500 mg/m^2^ qd 21d × 6 cycles)	④⑤	Chemotherapy alone	Not reported	**No**
Chen2010	67 (35/32)	39–71/35–68	Female	Ⅱ,Ⅲ	FFKS	(A): FFKS (20 mL + 0.9% 250 mLNSqd 14d) + (B)	(B): Docetaxel (75 mg/m^2^ qd 1d/20r × 6 cycles) + Epirubicin (50 mg/m^2^ qd 1d/20r × 6 cycles) + Cyclophosphamide (500 mg/m^2^ qd 21d × 6 cycles)	①⑩	Chemotherapy alone	Not reported	**No**
Zhai2014	123 (61/62)	26–70/27–73	Female	Ⅰ,Ⅱ,Ⅲ	FFKS	(A): FFKS (20 mL + 200 mL NS qd 21d × 6 cycles) + (B)	(B): Doxorubicin (50 mg/m^2^ + 50 mL NS 21d × 6 cycles) + Cyclophosphamide (50 mg/m^2^ + 50 mL NS 21d × 6 cycles) + 5-Fluorouracil (50 mg/m^2^ + 50 mL NS 21d × 6 cycles)	④⑤	Chemotherapy alone	Not reported	**No**
Feng2017	127 (63/64)	27–79/33–82	Female	Ⅱ,Ⅲ,Ⅳ	SQFZ	(A): SQFZ (250 mL qd 3d/18r × 4 cycles) + (B)	(B): Cyclophosphamide (500 mg/m^2^ 1d/20r × 4 cycles) + Doxorubicin (50 mg/m^2^ 1d/20r × 4 cycles) Fluorouracil (750 mg/m^2^ 1d/20r × 4 cycles)	①	Chemotherapy alone	Not reported	**No**
Guo2018	92 (46/46)	33–66/32–64	Female	Ⅰ,Ⅱ	SQFZ	(A): SQFZ (250 mL qd 21d × 6 cycles) + (B)	(B): Cyclophosphamide (500 mg/m^2^ 21d × 6 cycles) A66zithromycin (50 mg/m^2^ 21d × 6 cycles)+ 5-Fluorouracil (750 mg/m^2^ 21d × 6 cycles)	③④⑤⑥⑦⑧⑨	Chemotherapy alone	Not reported	**No**
Diao2018	94 (47/47)	31–37/33–73	Female	Ⅰ,II,Ⅲ	SQFZ	(A): SQFZ (250 mL 4d × 6 cycles) + (B)	(B): Cyclophosphamide (500 mg/m^2^ 1d/20r × 6 cycles)+ Epirubicin (100 mg/m^2^ 1d/20r × 6 cycles)+ Fluorouracil (500 mg/m^2^ 1d/20r × 6 cycles)+	③④⑤⑥	Chemotherapy alone	Not reported	**No**
Huang2008	60 (30/30)	24–68/26–66	Female	Ⅲ,Ⅳ	SQFZ	(A): SQFZ (250 mL qd 21d × 2 cycles) + (B)	(B): Cyclophosphamide (500 mg/m^2^ 2d/19r × 2cycles) + piroxicam (60 mg/m^2^ 1d/20rx 2 cycles) + 5-Fluorouracil (500 mg/m^2^ 2d/19r × 2 cycles)	③④⑤⑥	Chemotherapy alone	Not reported	**No**
Kuang2023	94 (47/47)	53.76 ± 4.17/52.37 ± 3.75	Female	Ⅰ,II,Ⅲ	FFKS	(A): FFKS (20 mL 6d/15r × 4 cycles) + (B)	(B): Doxorubicin (30 mg/m2 21d × 4cycles) + Cyclophosphamide (600 mg/m2 21d × 4 cycles) + Docetaxel (600 mg/m2 21d × 4 cycles) +	③⑥⑦⑧⑨	Chemotherapy alone	Not reported	**No**
Li2016	68 (34/34)	25–71/26–72	Female	Ⅲ,Ⅳ	FFKS	(A): FFKS (20 mL 14d/7r × 2 cycles) + (B)	(B): Paclitaxel (1d/20r × 2 cycles) + Doxorubicin (3d/18r × 2 cycles) +	①⑩	Chemotherapy alone	Not reported	**No**
Li2006	52 (32/20)	29–81/31–72	Female	Ⅰ,II,Ⅲ	AD	(A): AD (20 mL qd 28d) + (B)	(B): Cyclophosphamide (400–600 mg/m^2^ 2d/19r × 3 cycles) + 5-Fluorouracil (400–600 mg/m^2^ 2d/19r × 3 cycles) + Azithromycin (75–90 mg/m^2^ 1d/20r × 3 cycles) +	①	Chemotherapy alone	Not reported	**No**
Ye2019	90 (45/45)	27–65/28–63	Female	Ⅲ,Ⅳ	SQFZ	(A): SQFZ (250 mL qd 21d × 4 cycles) + (B)	(B): Cyclophosphamid (500 mg/m^2^ qd 21r × 4 cycles) + Azithromycin (50 mg/m^2^ qd 21r × 4 cycles) + 5-Fluorouracil (750 mg/m^2^ qd 21r × 4 cycles) +	③④⑤⑥	Chemotherapy alone	Not reported	**No**
Li2018	68 (34/34)	34–60/38–62	Female	II,Ⅲ	SQFZ	(A): SQFZ (3d/18r × 4 cycles) + (B)	(B): Docetaxel (75 mg/m^2^ qd 21r × 4 cycles)	①⑦⑧	Chemotherapy alone	Not reported	**No**
Lu2022	116 (58/58)	32–65/34–67	Female	Ⅰ,II,Ⅲ	FFKS	(A): FFKS (30 mL + 0.9% 250 mL NS qd 5d)/2r × 18 weeks + (B)	(B): Ifosfamide (500 mg/m^2^ 1r/20r × 6 cycles) + Fluorouracil (5000 mg/m^2^ 1r/20r × 6 cycles) + Epirubicin (80 mg/m^2^ 1r/20r × 6 cycles) +	③⑥	Chemotherapy alone	Not reported	**No**
Pan2011	160 (80/80)	42–58	Female	Ⅲ,Ⅳ	HCS	(A): HCS (20 mL qd 14d × 2 cycles) + (B)	(B): Cyclophosphamide (500 mg/m^2^ 2d/19r × 2 cycles) +Pirarubicin (40 mg/m^2^ 1d/20r × 2 cycles) + 5-Fluorouracil (400 mg/m^2^ 21rx 2 cycles)	①	Chemotherapy alone	Not reported	**No**
Qian2011	60 (30/30)	31–65	Female	Ⅲ,Ⅳ	HQ	(A): HQ (40 mL + 5%GLU 1d/20r) + (B)	(B): Gemcitabine (50 mg/m^2^ 21d) + Paclitaxel (175 mg/m^2^ 21d)	①	Chemotherapy alone	Not reported	**No**
Wang2019	94 (47/47)	32–68	Female	II,Ⅲ,Ⅳ	FFKS	(A): FFKS (20 mL + 5% 200 mL NS 10d/46r × 2 cycles) + (B)	(B): Epirubicin (70 mg/m^2^ 6d/50r × 2 cycles) + Docetaxel (75 mg/m^2^ 42d/14rx 2 cycles)	③④⑤⑥⑦⑧⑨⑩	Chemotherapy alone	Not reported	**No**
Ren2005	100 (60/40)	20–68	Female	II,Ⅲ	AD	(A): AD (80 mL + 0.9% 500 mL NS or 10% 500 mLGLU × 2 cycles + (B)	(B): Cyclophosphamide (600 mg/m^2^ 2d/19r × 2 cycles) + Methotrexate (30 mg/m^2^ 1d/20r × 2 cycles) + 5-Fluorouracil (500 mg/m^2^ 2d/19r × 2 cycles)	①⑩	Chemotherapy alone	Not reported	**No**
Wang2018	106 (53/53)	45.81 ± 7.84/46.18 ± 7.54	Female	II,Ⅲ	KLT	(A): KLT (200 mL qw 18 weeks) + (B)	(B): Cyclophosphamide (600 mg/m^2^ qw 21d × 6 cycles) + Doxorubicin (600 mg/m^2^ qw 21d × 6 cycles)	③④⑤⑥⑦⑨⑩	Chemotherapy alone	Not reported	**No**
Huang2012	40 (20/20)	33–75	Female	Ⅲ,Ⅳ	FFKS	(A): FFKS (20 mL 14d/7r × 2 cycles) + (B)	(B): Paclitaxel (135 mg/m^2^ 1d/20r × 2 cycles) + Doxorubicin (50 mg/m^2^ 3d/18r × 2 cycles)	①	Chemotherapy alone	Not reported	**No**
Wang2011	59 (30/29)	27–71	Female	Ⅰ,II,Ⅲ	HQ	(A): HQ (50 mL + 10% 500 mLGLU 10r × 2 cycles) + (B)	(B): Cyclophosphamide (500 mg/m^2^ qd 21r × 2 cycles) + Docetaxel (75 mg/m^2^ qd 21r × 2 cycles) + Epirubicin (50 mg/m^2^ qd 21r × 2 cycles)	⑩	Chemotherapy alone	Not reported	**No**
Fu2015	120 (60/60)	32–61/31–65	Female	Ⅰ,II,Ⅲ,Ⅳ	KLT	(A): KLT (200 mL 14d/7r × 6 cycles) + (B)	(B): Cyclophosphamide (500 mg/m^2^ 1d/20r × 6 cycles) + Doxorubicin (50 mg/m^2^ 1d/20r × 6 cycles) + Fuorouracil (50 mg/m^2^ 2d/20r × 6 cycless)	①⑦⑨	Chemotherapy alone	Not reported	**No**
Xu2017	104 (52/52)	42.4 ± 4.5/43.7 ± 5.3	Female	II,Ⅲ	FFKS	(A): FFKS (20 mL qd 21d × 2 cycles) + (B)	(B): Cyclophosphamide (21r × 6 cycles) + Doxorubicin (21r × 6 cycles) + Fuorouracil (21r × 6 cycles)	③④⑤	Chemotherapy alone	Not reported	**No**
Yang2019	110 (55/55)	31–58/32–56	Female	II,Ⅲ	FFKS	(A): FFKS (20 mL + 0.9% 250 mL NS 7d/14r × 6 cycles) + (B)	(B): Paclitaxel (135–175 mg/m^2^ qd 21d × 6 cycles) + Epirubicin (600 mg/m^2^ qd 21d × 6 cycles)	⑥	Chemotherapy alone	Not reported	**No**
She2017	384 (192/192)	20–77/19–76	Female	Ⅰ,II,Ⅲ	SQFZ	(A): SQFZ (250 mL qd 21d × 6 cycles) + (B)	(B): Docetaxel (75 mg/m^2^ 1d/20r × 6 cycles) + Docetaxel (60 mg/m^2^ 1d/20r × 6 cycles)	⑦⑧⑨	Chemotherapy alone	Not reported	**No**
Yang2013	60 (30/30)	41.50 ± 10.29	Female	Ⅲ,Ⅳ	FFKS	(A): FFKS (200 mL 14d/7r × 6 cycles) + (B)	(B): Paclitaxel (175 mg/m^2^ 21d)+ Epirubicin (180 mg/m^2^ 21d))	⑥⑩	Chemotherapy alone	Not reported	**No**
Pan2016	90 (45/45)	32–65/34–65	Female	Ⅲ,Ⅳ	KLT	(A): KLT (30 mL qd 21d/7r × 2 cycles) + (B)	(B): Cyclophosphamide (500 mg/m^2^ 1d/20r × 6 cycles) Doxorubicin (50 mg/m^2^ 1d/20r × 6 cycles) Fuorouracil (750 mg/m^2^ 1d/20r × 6 cycles)	③④⑤⑥⑦⑨	Chemotherapy alone	Not reported	**No**
Li2015	80 (43/37)	56–76/53–78	Female	Ⅰ,II,Ⅲ,Ⅳ	FFKS	(A): : FFKS (30 m + 0.9% 250 mL NS qd 7d/14r × 6 cycles) + (B)	(B): Paclitaxel (135–175 mg/m^2^ bid 21d × 6 cycles) Azithromycin (60 mg/m^2^ bid 21d × 6 cycles)	④⑤	Chemotherapy alone	Not reported	**No**
Zhang2015	130 (65/65)	43.2 ± 17.9	Female	Ⅰ,II	FFKS	(A): FFKS (12 mL + 200 mL NS) + (B)	(B): Doxorubicin (30–40 mg/m^2^ 1d/20r) Cyclophosphamide (600 mg/m^2^1d/20r)	②	Chemotherapy alone	Not reported	**No**
Zhu2021	90 (46/44)	33–67/34–68	Female	Ⅲ,Ⅳ	HQ	(A): HQ (40 mL qd 7d/14r × 3 cycles) + (B)	(B): Paclitaxel liposome (135 mg/kg 1d/20r × 3 cycles)	③④⑥	Chemotherapy alone	Not reported	**No**
Lu2018	100 (50/50)	46.11 ± 10.76	Female	Ⅲ,Ⅳ	SQFZ	(A): SQFZ (250 mL qd × 2 cycles) + (B)	(B): Cyclophosphamide (500 mg/m^2^ 1d × 2cycles) Doxorubicin (50 mg/m^2^ 1d × 2 cycles) Docetaxel (75 mg/m^2^ 1d × 2 cycles) NS (250 mL qd × 2 cycles)	③④⑤⑥	Chemotherapy alone	Not reported	**No**
Sun2023	92 (46/46)	31–72/32–74	Female	Ⅲ,Ⅳ	FFKS	(A): FFKS (20 mL qd 14d/7r × 4 cycles) + (B)	(B): Docetaxel (75 mg/m^2^ 1d/20r × 4 cycles) Pirarubicin (50 mg/m^2^ 20d × 4 cycles) Cyclophosphamide (600 mg/m^2^ 20d × 4 cycles)	①③④⑤⑥⑦⑧⑨	Chemotherapy alone	Not reported	**No**
Luo2023	86 (43/43)	61.87 ± 5.25/61.24 ± 5.19	Female	II,Ⅲ	SQFZ	(A): SQFZ (250 mL qd 21d × 3 cycles) + (B)	(B): Cyclophosphamide (500 mg/m^2^ 1d/20r × 3 cycles) Doxorubicin (50 mg/m^2^ 1d/20r × 3 cycles) Fuorouracil (600 mg/m^2^ 1d/20r × 3 cycles)	③④⑦⑧⑨	Chemotherapy alone	Not reported	**No**
Ma2011	63 (32/31)	28–63/30–62	Female	Ⅲ,Ⅳ	FFKS	(A): FFKS (20 mL qd 21d × 3 cycles) + (B)	(B): Docetaxel (75 mg/m^2^ 1d/20r × 3 cycles) Epirubicin (50 mg/m^2^ 1d/20r × 3 cycles) Cyclophosphamide (500 mg/m^2^ 1d/20r × 3 cycles)	②	Chemotherapy alone	Not reported	**No**
Wei2023	84 (42/42)	36–78/38–79	Female	II,Ⅲ,Ⅳ	SQFZ	(A): SQFZ (250 mL 3d/18r × 6 cycles) + (B)	(B): Docetaxel (75 mg/m^2^ 1d/20r × 6 cycles) Gemcitabine (1000 mg/m^2^ 2d/19r × 6 cycles)	④⑤⑥⑦⑧⑨	Chemotherapy alone	Not reported	**No**
Xie2011	60 (30/30)	36–65/35–60	Female	Ⅰ,II	SM	(A): SM (50 mL + 5% 250 mLGLU qd 14d/7r × 6 cycles) + (B)	(B): Docetaxel (750 mg/m^2^ 1d/20r × 6cycles) Doxorubicin (50 mg/m^2^ 1d/20r × 6 cycles) Cyclophosphamide (500 mg/m^2^ 1d/20r × 6 cycles)	③④⑤⑥	Chemotherapy alone	Not reported	**No**
Lao2011	60 (30/30)	31–62/28–60	Female	Ⅲ,Ⅳ	SM	(A): SM (40 mL + 0.15% 250 mLGLU qd 14d × 2 cycles) + (B)	(B): Cyclophosphamide (500 mg/m^2^ 2d/26r × 2 cycles) Doxorubicin (50–60 mg/m^2^ 1d/27r × 2 cycles) 5-Fluorouracil (500 mg/m^2^ 3d/25r × 2 cycles)	②③④⑤⑥	Chemotherapy alone	Not reported	**No**
Chen2010	60 (30/30)	35–59/32–63	Female	Ⅲ,Ⅳ	SM	(A): SM (100 mL qd 21d/15r × 3 cycles) + (B)	(B): Cyclophosphamide (500 mg/m^2^ 2d/19–26r × 3 cycles) Doxorubicin (30–40 mg/m^2^ 1d/20–27r × 3 cycles) 5-Fluorouracil (500 mg/m^2^ 3d/18–25r × 3 cycles)	②③④⑤⑥	Chemotherapy alone	Not reported	**No**
Huang2009	60 (30/30)	23–61/22–64	Female	Ⅲ,Ⅳ	SM	(A): SM (60 mL + 5% 250 mLGLU qd 21d × 2 cycles) + (B)	(B): Cyclophosphamide (500 mg/m^2^ 2d/19r × 2 cycles) Pirarubicin (60 mg/m^2^ 1d/20r × 2 cycles) 5-Fluorouracil (75 mg/m^2^ 2d/19r × 2 cycles)	③④⑤⑥	Chemotherapy alone	Not reported	**No**
Chen2022	70 (35/35)	29–68/32–69	Female	II,Ⅲ	KA	(A): KA (40 mL qd 21d × 2 cycles) + (B)	(B): 5-Fluorouracil (500 mg/(m^2^·d) over 6 h,21d × 2 cycles) Docetaxel (60–75 mg/m^2^ 21d × 2 cycles)	①	Chemotherapy alone	Not reported	**No**
Qiu2016	120 (60/60)	32–64/35–64	Female	II,Ⅲ	KA	(A): KA (40 mL + NS qd 21d) + (B)	(B): Cyclophosphamide (600 mg/m^2^ 1d/20r × 4 cycles) Azithromycin (80–100 mg/m^2^ 1d/20r × 2 cycles)	①③④⑤⑩	Chemotherapy alone	Not reported	**No**
Shi2017	60 (30/30)	25–63/26–65	Female	Ⅰ,II,Ⅲ	KA	(A): KA (30 mL qd 15/6d × 3 cycles) + (B)	(B): Doxorubicin (50 mg/m^2^ +50 mL NS 21d × 3 cycles) Cyclophosphamide (500 mg/m^2^+30 mL NS 21d × 3 cycles) 5-Fluorouracil (500 mg/m^2^+500 mL NS 21d × 3 cycles)	④⑤	Chemotherapy alone	Not reported	**No**
Zhang2012	93 (47/46)	32–69/34–68	Female	II,Ⅲ	KA	(A): KA (40–60 mL + 5% 250 mL-500 mLGLU or 0.9% 250 mL-500 mL NS qd 30d) + (B)	(B): Cyclophosphamide (0.6 g/m^2^ qd 21d × 2 cycles) Doxorubicin (60 mg/m^2^qd 21d × 2 cycles)	⑩	Chemotherapy alone	Not reported	**No**
Zheng 2021	58 (29/29)	36–68/37–70	Female	Ⅲ,Ⅳ	KA	(A): KA (40 mL qd 10d × 8 cycles) + (B)	(B): Cyclophosphamide (600 mg/m^2^ 21 d × 4 cycles) Epirubicin (80 mg/m^2^ 21 d × 4 cycles) Paclitaxel (175 mg/m^2^ From the fifth cycle 21d × 4 cycles)	③④⑥	Chemotherapy alone	Not reported	**No**

### 3.5 Quality assessment

**FIGURE 2:**
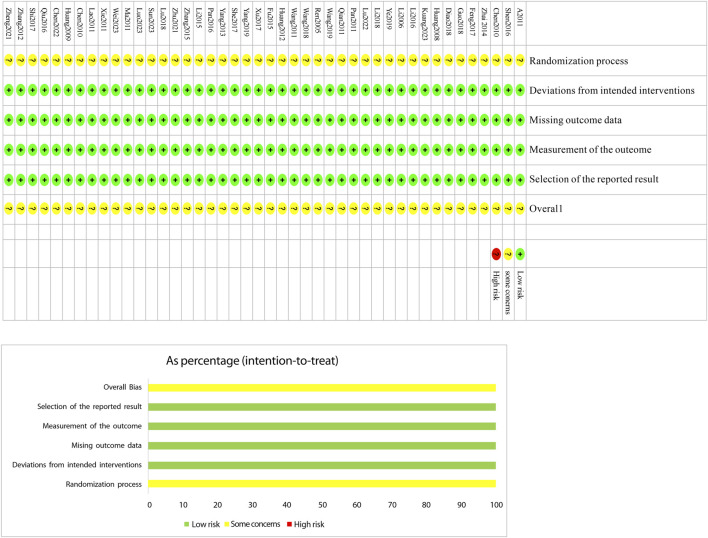
Assessment Figure of risk of bias.

The Cochrane Risk of Bias tool (RoB 2.0) (Tsou and Treadwell, 2016) was used to assess the included studies across six domains: bias due to the randomization process, bias due to deviations from intended interventions, bias due to missing outcome data, bias due to measurement of the outcome, bias due to selective reporting of results, and bias from other sources. Two investigators independently evaluated each study across these six domains, categorizing them as “low risk,” “high risk,” or “some concerns.” In cases of disagreement, discrepancies were resolved through discussion or by consulting a third investigator. The results of the assessment were presented in a risk-of-bias graph (Figure 2).

### 3.6 Statistical analysis

This study employed R Studio and STATA 17 software for network meta-analysis. The final included randomized controlled trials (RCTs) were analyzed using the “gemtc” and “coda” packages in R Studio version 4.2.1. The simulation analysis employed four Markov chains initialized at 2.5 with a thinning interval of 1. A burn-in period of 50,000 iterations was implemented for model annealing, followed by 20,000 iterations to achieve model convergence (Bois, 2013; Dias et al., 2012). The deviance information criterion (DIC) of the iteration results was then compared with that of the inconsistency model. A difference of less than 5 indicated data consistency, suggesting no significant discrepancy between the NMA results and direct comparisons. The “ranks” command was used to rank the effect sizes, generating a ranking table, while the “SUCRA” command calculated the surface under the cumulative ranking curve (SUCRA) values for cumulative probability ranking within the table ( Rucker and Schwarzer, 2015 ). The “tb” command processed data to produce a league table comparing relative effects among different interventions. Relative risk ratios (RR) were reported for dichotomous data, and weighted mean differences (WMD) were reported for continuous data. Both measures were presented with 95% confidence intervals (CI) to estimate differences between interventions. Network diagrams created using the “networkplot” command in STATA 17 to illustrate direct and indirect comparisons between interventions. Funnel plots were generated via “network convert pairs” to assess publication bias and small-study effects. Rank probability plots to estimate intervention rankings across different outcome measures. Toaccount for heterogeneity between trials, a Bayesian hierarchical random-effects model was initially employed for comparing varioustreatment options for BC.

## 4 Results

### 4.1 Search results

The brief process of screening and inclusion: A total of 9,796 articles were identified from eight databases according to the search strategy described in Section 3.1. Using the EndNote reference manager, 3,497 articles were removed by machine-based duplicate checking, and an additional 3,096 duplicate articles were manually removed. Subsequently, articles were excluded based on the following criteria: trials (712), reviews (1,527), case reports (54), other irrelevant topics (796), and study registrations (17). Among the remaining 97 articles, full - text reading was conducted. A further 51 articles were excluded due to low quality of the literature, non - conforming data, and missing data. Additionally, 2 articles were excluded due to flawed experimental design. Finally, 44 articles were included. For the detailed process, please refer to Figure 1.

### 4.2 Characteristics of the included studies

All 44 included studies (Atikan & Ahmatjan, 2011; Chen, 2010; Chen et al., 2010; Chen, 2022; Diao et al., 2018; Feng, 2017; Fu et al., 2015; Guo and Hu, 2018; Huang et al., 2009; Huang et al., 2012; Huang et al., 2008; Kuang, 2023; Lao et al., 2011; Li and Liu, 2018; Li and Zhang, 2015; Li, 2016; Li and Gong, 2006; Lu, 2022; Lu et al., 2018; Luo, 2023; Ma et al., 2011; Pan, 2011; Pan et al., 2016; Qian, 2011; Qiu, 2016; Ren and Zhang, 2005; She et al., 2017; Shen, 2016; Shi et al., 2017; Sun, 2023; Wang and Chu, 2018; Wang and Ren, 2019; Wang et al., 2011; Wei, 2023; Xie et al., 2011; Xu, 2017; Yang, 2019; Yang, 2013; Ye et al., 2019; Zhai, 2014; Zhang, 2012; Zhang, 2015; Zheng, 2021; Zhu, 2021)were conducted in China, involving a total of 3,982 patients. Among them, 2,048 patients were in the experimental groups, and 1,934 patients were in the control groups. Eight types of Chinese herbal injections (CHIs) were evaluated, including SQFZ plus chemotherapy (11 RCT), FFKS plus chemotherapy (15 RCT), Cinobu facini Injection plus chemotherapy (1 RCT), KA plus chemotherapy (5 RCT), HQ plus chemotherapy (3 RCT), SM plus chemotherapy (4 RCT), KLT plus chemotherapy (2 RCT), and AD plus chemotherapy (2 RCT). All 44 included studies had control groups that received chemotherapy alone, without placebo controls. Regarding the implementation of blinding, none of the studies reported the use of blinding. All the included studies were conducted in China and were single-center studies. The basic characteristics of the included literature are presented in Table 2.

### 4.3 Quality assessment of the selected studies

The risk-of-bias assessment results for the 44 included studiesare shown in Figure 2. In terms of bias in randomization, all 44 studies were assessed as having a potential risk due to the lackof a description of randomization and blinding. Allstudies were assessed as having a low risk ofbias in terms ofdeviations from established interventions, missing data onoutcomes, measurements and selective reporting. No othersources of bias were found in any of the included studies, whichwere assessed to be at low risk. Taken together, the risk of bias in theincluded literature was low.

### 4.4 Outcome measures

#### 4.4.1 Primary outcome measure

##### 4.4.1.1 Efficacy

**FIGURE 3:**
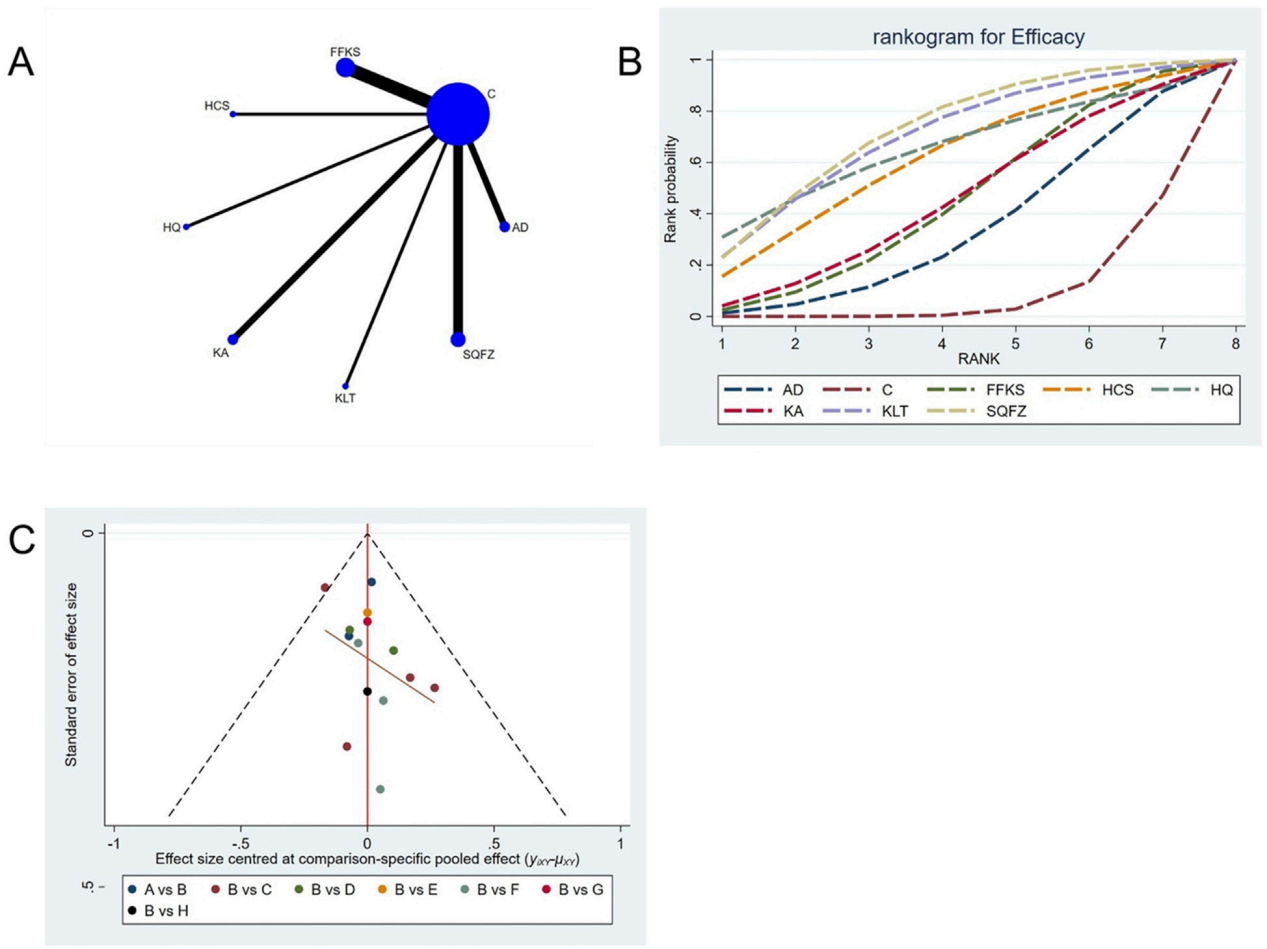
Network meta-analysis results for Efficacy. **(A)** Network plot **(B)** Cumulative probability ranking curve of different interventions. **(C)** Funnel plot. AD, Aidi Injection; FFKS, Compound Sophora Injection; HCS, Cinobu facini Injection; HQ, Huangqi Injection; KA, Kangai Injection; KLT, Kanglaite Injection; SQFZ, Shenqifuzheng Injection. T, Treatment; C, Control group.

Efficacy is the best indicator for evaluating cancer treatment outcomes. For this outcome measure, a total of 14 randomized controlled trials (RCTs) evaluating 7 Chinese herbal injections (CHls) were included. The network structure of these interventions is presented in Figure 3A (The size of the nodes represents the sample size, and the thickness of the connecting lines represents the number of direct comparisons). Among them, SQFZ combined with the conventional treatment group had the largest sample size, while the FFKS was investigated in the greatest number of studies. The deviance information criterion (DIC) difference was 0.09452. Pairwise comparisons demonstrated statistically significant superiority of SQFZ over the control group in 3 studies (RR = 1.35, 95% CI: 1.01, 1.85) (see Table 3). The rank probability analysis (Figure 3B) (The size of the nodes represents the sample size, the thickness of the connecting lines represents the number of direct comparisons, and the SUCRA value represents the area under the curve) revealed the top treatments: SQFZ (SUCRA: 72.15%). The funnel plot reflects whether there is potential bias (Figure 3C) (with the horizontal axis representing the risk ratio [RR] and the vertical axis representing the standard error; asymmetry of the funnel plot indicates the presence of bias). As shown in the figure, there is potential bias.

**TABLE 3:** Efficacy league table.

AD	0.91 (0.69, 1.15)	1.05 (0.76, 1.52)	1.16 (0.72, 1.82)	1.21 (0.67, 2.19)	1.06 (0.71, 1.54)	1.21 (0.77, 1.89)	1.23 (0.82, 1.81)
1.1 (0.87, 1.45)	C	1.16 (0.95, 1.52)	1.28 (0.87, 1.88)	1.34 (0.8, 2.32)	1.17 (0.87, 1.57)	1.34 (0.93, 1.96)	1.35 (1.01, 1.85)
0.95 (0.66, 1.31)	0.86 (0.66, 1.05)	FFKS	1.1 (0.68, 1.67)	1.15 (0.63, 2.04)	1 (0.67, 1.42)	1.16 (0.72, 1.74)	1.16 (0.78, 1.67)
0.87 (0.55, 1.39)	0.78 (0.53, 1.14)	0.91 (0.6, 1.47)	HCS	1.05 (0.55, 2.04)	0.91 (0.56, 1.49)	1.05 (0.62, 1.79)	1.06 (0.66, 1.74)
0.83 (0.46, 1.49)	0.75 (0.43, 1.26)	0.87 (0.49, 1.58)	0.96 (0.49, 1.83)	HQ	0.87 (0.47, 1.59)	1 (0.52, 1.91)	1.02 (0.54, 1.86)
0.95 (0.65, 1.41)	0.86 (0.64, 1.14)	1 (0.71, 1.49)	1.09 (0.67, 1.77)	1.14 (0.63, 2.13)	KA	1.15 (0.72, 1.85)	1.16 (0.76, 1.78)
0.82 (0.53, 1.3)	0.75 (0.51, 1.07)	0.86 (0.57, 1.38)	0.95 (0.56, 1.61)	1 (0.52, 1.93)	0.87 (0.54, 1.4)	KLT	1.01 (0.63, 1.63)
0.81 (0.55, 1.22)	0.74 (0.54, 0.99)	0.86 (0.6, 1.28)	0.94 (0.58, 1.52)	0.98 (0.54, 1.84)	0.86 (0.56, 1.31)	0.99 (0.61, 1.59)	SQFZ

The ones marked in red are statistically significant.

##### 4.4.1.2 KPS

**FIGURE 4:**
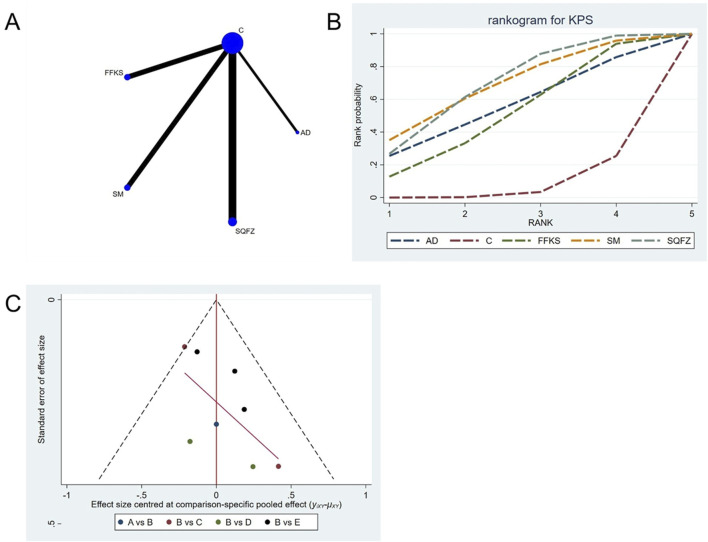
Network meta-analysis results for Efficacy. **(A)** Network plot **(B)** Cumulative probability ranking curve of different interventions. **(C)** Funnel plot. AD, Aidi Injection; FFKS, Compound Sophora Injection; SM, Shenmai Injection; SQFZ, Shenqifuzheng Injection. T, Treatment; C, Control group.

Quality of life, as assessed by the Karnofsky Performance Status (KPS), is one of the primary indicators for evaluating cancer treatment outcomes. For this outcome measure, a total of 8 randomized controlled trials (RCTs) evaluating 4 Chinese herbal injections (CHIs) were included. The network structure of these interventions is presented in (Figure 4A). Among them, SQFZ combined with the conventional treatment group had the largest sample size and was investigated in the greatest number of studies compared to the control group. The deviance information criterion (DIC) difference was −0.18342. Pairwise comparisonsdemonstrated statistically significant superiority over the control group for:SQFZin 3 studies (RR = 1.78, 95% CI: 1.09, 3.68) (see Table 4). The rank probability analysis (Figure 4B) revealed the top treatments:SQFZ (SUCRA: 68.73%). The funnel plot reflects whether there is potential bias (Figure 4C). As shown in the figure, suggesting a low risk of publication bias for this indicator.

**TABLE 4:** KPS league table.

AD	0.62 (0.22, 1.75)	0.94 (0.29, 3.55)	1.15 (0.32, 4.31)	1.12 (0.36, 4.09)
1.6 (0.57, 4.58)	C	1.5 (0.81, 3.36)	1.84 (0.86, 4.16)	1.78 (1.09, 3.68)
1.06 (0.28, 3.48)	0.66 (0.3, 1.24)	FFKS	1.21 (0.4, 3.33)	1.19 (0.48, 3.06)
0.87 (0.23, 3.17)	0.54 (0.24, 1.16)	0.82 (0.3, 2.51)	SM	0.98 (0.38, 2.83)
0.89 (0.24, 2.75)	0.56 (0.27, 0.92)	0.84 (0.33, 2.09)	1.02 (0.35, 2.61)	SQFZ

The ones marked in red are statistically significant.

#### 4.4.2 Immune function assessment indicators

The immunological indices consist of four T-cell surface markers—CD3^+^, CD4^+^, CD8^+^, and the CD4^+^/CD8^+^ ratio (Wen et al., 2023)—whose maintenance within reference intervals indicates adequate overall immune reserve and the absence of substantial immunological imbalance, thereby exerting a prognostic influence.

##### 4.4.2.1 Immunological function: CD3^+^ T-cell analysis

**FIGURE 5:**
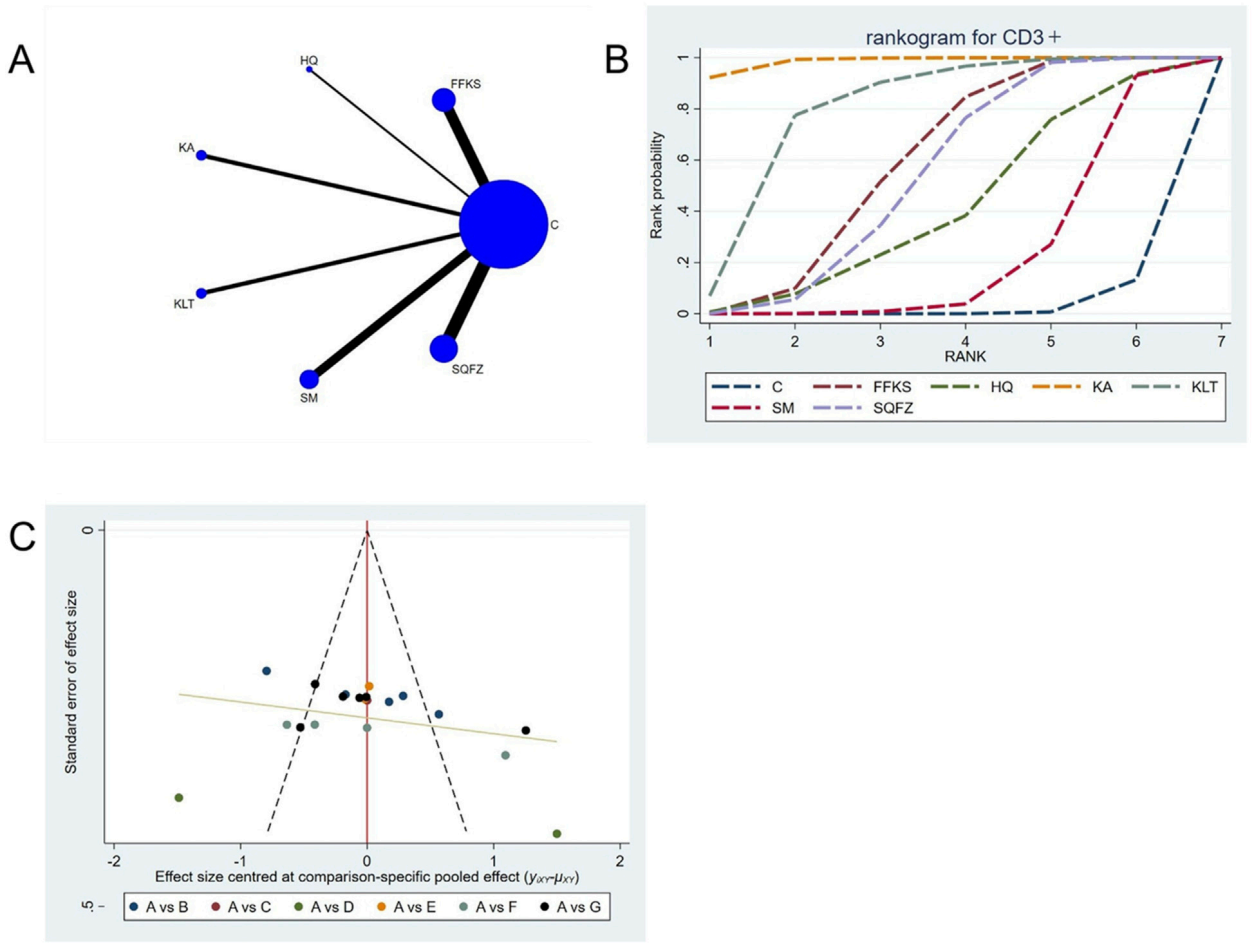
Network meta-analysis results for Efficacy. **(A)** Network plot **(B)** Cumulative probability ranking curve of different interventions. **(C)** Funnel plot. FFKS, Compound Sophora Injection; HQ, Huangqi Injection; KA, Kangai Injection; KLT, Kanglaite Injection; SM, Shenmai Injection; SQFZ, Shenqifuzheng Injection. T, Treatment; C, Control group.

CD3^+^ constitutes an integral component of the T-cell receptor (TCR) complex and is indispensable for antigen recognition and subsequent T-cell signal transduction (Lee et al., 2019). It mediates direct cytotoxicity against neoplastic cells (Lee et al., 2019). Accumulating evidence demonstrates that an elevated CD3^+^ level is significantly associated with improved prognosis in patients with breast cancer (Tsiatas et al., 2018). For this outcome measure, a total of 20 randomized controlled trials (RCTs) evaluating 6 Chinese herbal injections (CHIs) were included. The network structure of these interventions is presented in (Figure 5A). Among them, the Shenqifuzheng Injection (SQFZ) combined with the conventional treatment group had the largest sample size and was investigated in the greatest number of studies compared to the control group. The deviance information criterion (DIC) difference was 0.11962. Pairwise comparisons demonstrated statistically significant superiority over the control group for the following treatments (see Table 5): SQFZ in 6 studies (SMD = 8.47, 95% CI: 4.79, 12.25), FFKS in 5 studies (SMD = 7.85, 95% CI: 4.41, 11.37), KA in 2 studies (SMD = 18.49, 95% CI: 12.74, 24.17), KLT in 1 study (SMD = 12.3, 95% CI: 5.95, 18.69). The rank probability analysis (Figure 5B) revealed the top treatments:Kangai Injection (SUCRA: 98.56%). The funnel plot reflects whether there is potential bias (Figure 5C), the funnel plot shows a left-skewed distribution (with a dense concentration in the area where the effect size is less than −5), indicating the presence of publication bias for the CD3^+^ indicator.

**TABLE 5:** CD3+ league table.

C	8.47 (4.79, 12.15)	5.97 (−2.05, 13.94)	18.49 (12.74, 24.17)	12.3 (5.95, 18.69)	3.06 (−1.22, 7.19)	7.85 (4.41, 11.37)
−8.47 (−12.15, −4.79)	FFKS	−2.5 (−11.36, 6.28)	10.02 (3.22, 16.78)	3.82 (−3.51, 11.19)	−5.42 (−11.08, 0.12)	−0.62 (−5.62, 4.49)
−5.97 (−13.94, 2.05)	2.5 (−6.28, 11.36)	HQ	12.53 (2.67, 22.31)	6.34 (−3.86, 16.54)	−2.9 (−12.04, 6.14)	1.89 (−6.79, 10.69)
−18.49 (−24.17, −12.74)	−10.02 (−16.78, −3.22)	−12.53 (−22.31, −2.67)	KA	−6.19 (−14.71, 2.37)	−15.44 (−22.55, −8.38)	−10.65 (−17.22, −3.87)
−12.3 (−18.69, −5.95)	−3.82 (−11.19, 3.51)	−6.34 (−16.54, 3.86)	6.19 (−2.37, 14.71)	KLT	−9.25 (−16.95, −1.64)	−4.44 (−11.72, 2.83)
−3.06 (−7.19, 1.22)	5.42 (−0.12, 11.08)	2.9 (−6.14, 12.04)	15.44 (8.38, 22.55)	9.25 (1.64, 16.95)	SM	4.8 (−0.56, 10.35)
−7.85 (−11.37, −4.41)	0.62 (−4.49, 5.62)	−1.89 (−10.69, 6.79)	10.65 (3.87, 17.22)	4.44 (−2.83, 11.72)	−4.8 (−10.35, 0.56)	SQFZ

The ones marked in red are statistically significant.

##### 4.4.2.2 Immunological function: CD4^+^ T-cell analysis

**FIGURE 6:**
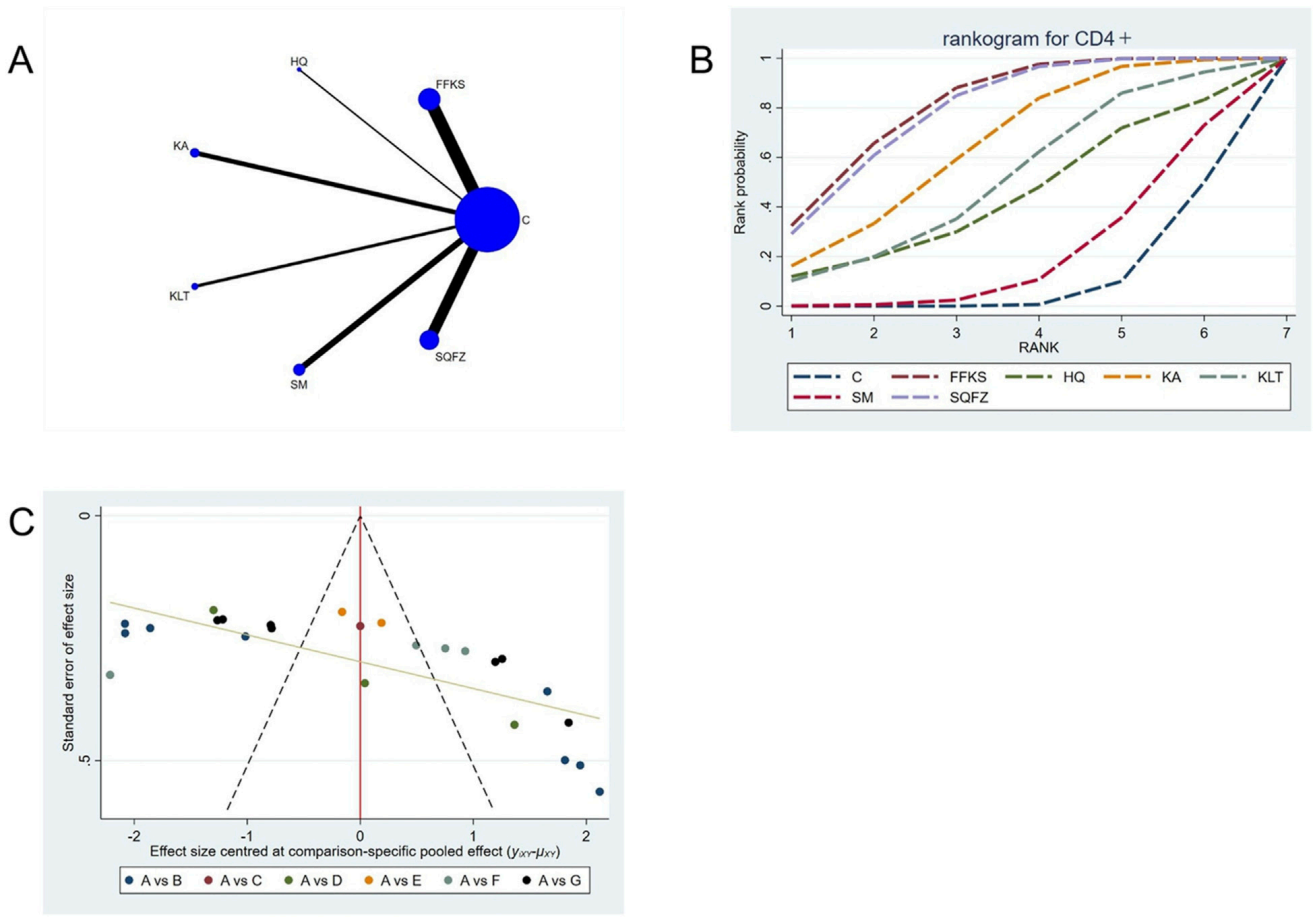
Network meta-analysis results for Efficacy. **(A)** Network plot **(B)** Cumulative probability ranking curve of different interventions. **(C)** Funnel plot. FFKS, Compound Sophora Injection; HQ, Huangqi Injection; KA, Kangai Injection; KLT, Kanglaite Injection; SM, Shenmai Injection; SQFZ, Shenqifuzheng Injection. T, Treatment; C, Control group.

CD4^+^ is a transmembrane glycoprotein predominantly expressed on the surface of helper T lymphocytes (CD4^+^ T cells) (Tsiatas et al., 2018). Clinical evidence has demonstrated that CD4^+^ T cells may facilitate tumour progression through the secretion of inhibitory cytokines that suppress the antitumour type-1 T-helper (Th1) immune response (Niwinska and Olszewski, 2021). For this outcome measure, a total of 25 randomized controlled trials (RCTs) evaluating 6 Chinese herbal injections (CHIs) were included. The network structure of these interventions is presented in Figure 6A. Among them, the FFKS combined with the conventional treatment group had the largest sample size and was investigated in the greatest number of studies compared to the control group. The deviance information criterion (DIC) difference was 0.0022. Pairwise comparisons demonstrated statistically significant superiority over the control group for the following treatments (see Table 6), FFKS in 8 studies (SMD = 10.05, 95% CI: 5.94, 14.18),SQFZ in 7 studies (SMD = 9.82, 95% CI: 5.33, 14.24),KAin 3 studies (SMD = 8.04, 95% CI: 1.39, 14.76). The rank probability analysis (Figure 6B) revealed the toptreatments: FFKS (SUCRA: 80.63%). The funnel plot reflects whether there is potential bias (Figure 6C), the funnel plot is sparsely distributed in the positive effect area (right side) (with no data points where the standard error is greater than 1), showing a right-skewed distribution, which may suggest the presence of publication bias.

**TABLE 6:** CD4+ league table.

C	10.05 (5.94, 14.18)	4.81 (−6.71, 16.4)	8.04 (1.39, 14.76)	6.08 (−2.49, 14.63)	1.3 (−4.54, 7.2)	9.82 (5.33, 14.24)
−10.05 (−14.18, −5.94)	FFKS	−5.24 (−17.5, 7.1)	−2.02 (−9.85, 5.86)	−3.97 (−13.52, 5.45)	−8.76 (−15.91, −1.54)	−0.23 (−6.33, 5.8)
−4.81 (−16.4, 6.71)	5.24 (−7.1, 17.5)	HQ	3.23 (−10.16, 16.62)	1.26 (−13.12, 15.63)	−3.52 (−16.46, 9.45)	5.01 (−7.45, 17.33)
−8.04 (−14.76, −1.39)	2.02 (−5.86, 9.85)	−3.23 (−16.62, 10.16)	KA	−1.98 (−12.87, 8.84)	−6.75 (−15.61, 2.13)	1.78 (−6.33, 9.75)
−6.08 (−14.63, 2.49)	3.97 (−5.45, 13.52)	−1.26 (−15.63, 13.12)	1.98 (−8.84, 12.87)	KLT	−4.77 (−15.09, 5.64)	3.76 (−5.89, 13.42)
−1.3 (−7.2, 4.54)	8.76 (1.54, 15.91)	3.52 (−9.45, 16.46)	6.75 (−2.13, 15.61)	4.77 (−5.64, 15.09)	SM	8.51 (1.11, 15.84)
−9.82 (−14.24, −5.33)	0.23 (−5.8, 6.33)	−5.01 (−17.33, 7.45)	−1.78 (−9.75, 6.33)	−3.76 (−13.42, 5.89)	−8.51 (−15.84, −1.11)	SQFZ

The ones marked in red are statistically significant.

##### 4.4.2.3 Immunological function: CD8^+^ T-cell analysis

**FIGURE 7:**
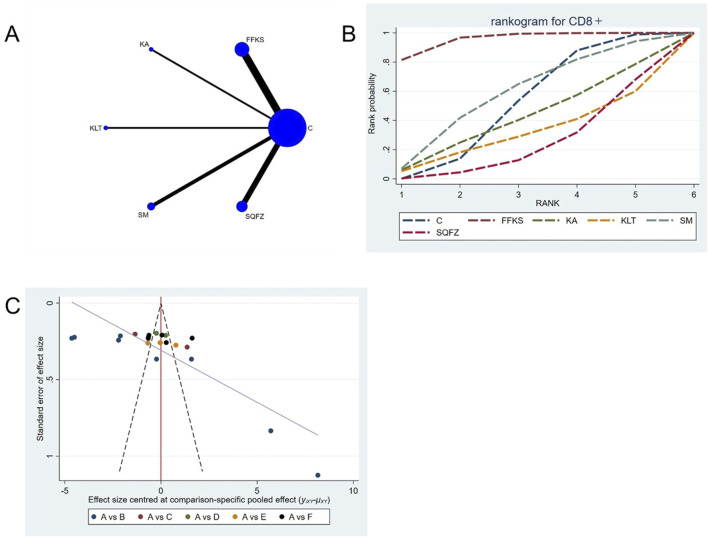
Network meta-analysis results for Efficacy. **(A)** Network plot **(B)** Cumulative probability ranking curve of different interventions. **(C)** Funnel plot. FFKS, Compound Sophora Injection; KA, Kangai Injection; KLT, Kanglaite Injection; SM, Shenmai Injection; SQFZ, Shenqifuzheng Injection. T, Treatment; C, Control group.

CD8^+^ is a transmembrane glycoprotein predominantly expressed on the surface of cytotoxic T lymphocytes (CD8^+^+T cells) (Steele et al., 2018). These CD8^+^ T cells are regarded as the principal effectors of antitumor immunity, as they mediate tumor elimination through the recognition of tumor-associated antigens and subsequent direct cytotoxicity via the secretion of perforin and granzyme B (Li et al., 2025). For this outcome measure, a total of 22 randomized controlled trials (RCTs) evaluating 5 Chinese herbal injections (CHIs) were included. The network structure of these interventions is presented in (Figure 7A). Among them, FFKS combined with the conventional treatment group had the largest sample size and was investigated in the greatest number of studies compared to the control group. The deviance information criterion (DIC) difference was 0.025. Pairwise comparisons demonstrated statistically significant superiority over the control group for FFKS in 8 studies (SMD = 7.46, 95% CI: 2.11, 12.76) and SQFZ in 6 studies (SMD = −10.79,95% CI:-19.06, −2.63) (see Table 7). The rank probability analysis (Figure 7B) revealed the top treatments: FFKS (SUCRA: 95.48%). The funnel plot reflects whether there is potential bias (Figure 7C), the funnel plot is asymmetrically distributed, with dense clustering on the left side, suggesting the presence of bias.

**TABLE 7:** CD8+ league table.

C	7.46 (2.11, 12.76)	−1.22 (−11.81, 9.37)	−3.27 (−16.09, 9.53)	1.1 (−6.5, 8.71)	−3.33 (−9.64, 2.83)
−7.46 (−12.76, −2.11)	FFKS	−8.65 (−20.59, 3.2)	−10.72 (−24.54, 3.21)	−6.35 (−15.6, 2.98)	−10.79 (−19.06, −2.63)
1.22 (−9.37, 11.81)	8.65 (−3.2, 20.59)	KA	−2.08 (−18.67, 14.56)	2.33 (−10.71, 15.38)	−2.12 (−14.44, 10.19)
3.27 (−9.53, 16.09)	10.72 (−3.21, 24.54)	2.08 (−14.56, 18.67)	KLT	4.37 (−10.43, 19.31)	−0.05 (−14.38, 14.12)
−1.1 (−8.71, 6.5)	6.35 (−2.98, 15.6)	−2.33 (−15.38, 10.71)	−4.37 (−19.31, 10.43)	SM	−4.44 (−14.34, 5.36)
3.33 (−2.83, 9.64)	10.79 (2.63, 19.06)	2.12 (−10.19, 14.44)	0.05 (−14.12, 14.38)	4.44 (−5.36, 14.34)	SQFZ

The ones marked in red are statistically significant.

##### 4.4.2.4 Immunological function: CD4+/CD8+ T-cell analysis

**FIGURE 8:**
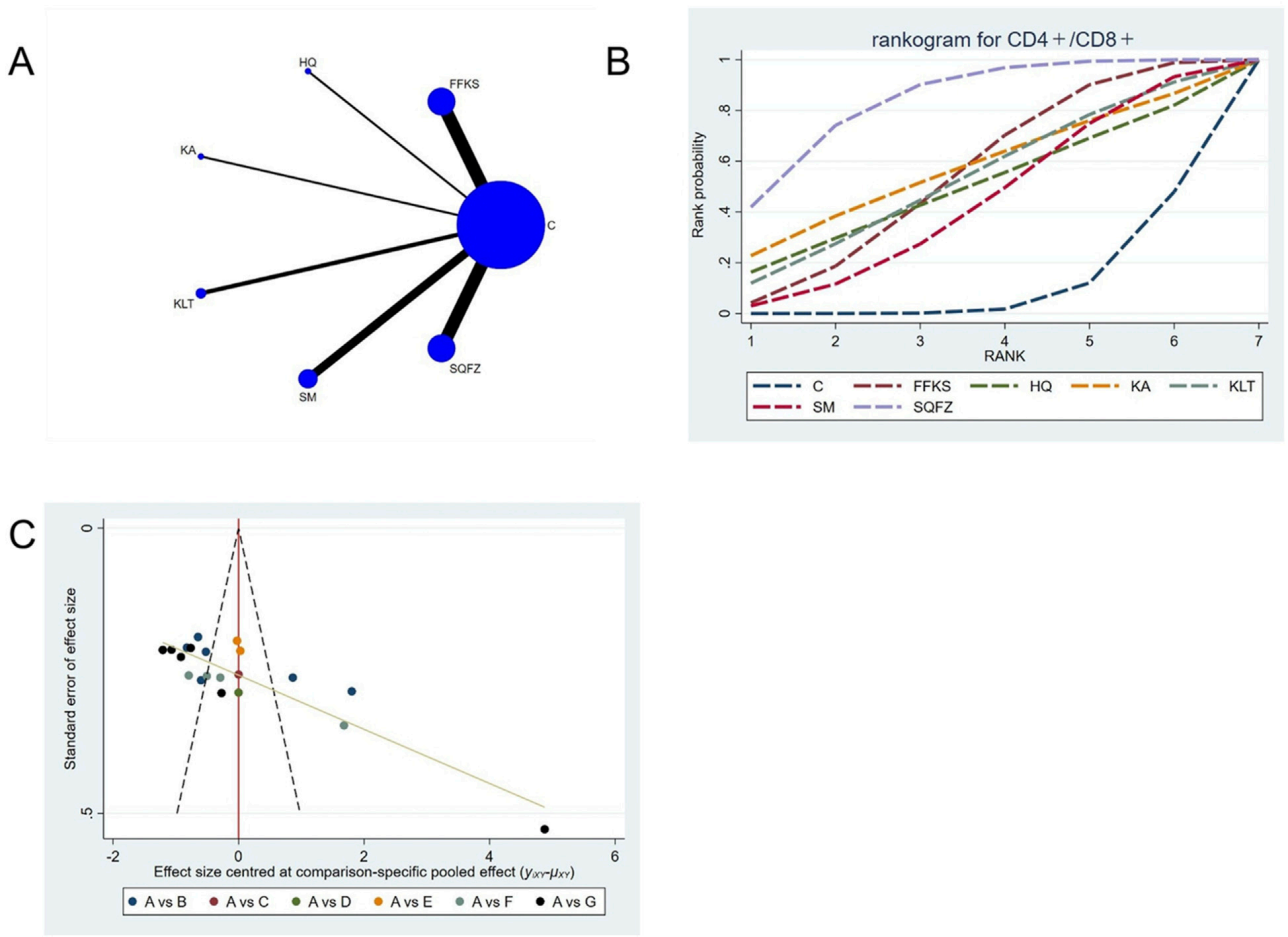
Network meta-analysis results for Efficacy. **(A)** Network plot **(B)** Cumulative probability ranking curve of different interventions. **(C)** Funnel plot. FFKS, Compound Sophora Injection; HQ, Huangqi Injection; KA, Kangai Injection; KLT, Kanglaite Injection; SM, Shenmai Injection; SQFZ, Shenqifuzheng Injection. T, Treatment; C, Control group.

The CD4^+^/CD8^+^ ratio is employed as a quantitative indicator for evaluating the homeostatic status of cellular immunity (Yu et al., 2015). For this outcome measure, a total of 20 randomized controlled trials (RCTs) evaluating 6 Chinese herbal injections (CHIs) were included. The network structure of these interventions is presented in (Figure 8A). Among them, FFKS and SM combined with the conventional treatment groups included the same number of studies (6 RCTs each), with FFKS being investigated in the greatest number of studies compared to the control group. The deviance information criterion (DIC) difference was 0.05248. Pairwise comparisons demonstrated statistically significant superiority over the control group for FFKS in 6 studies (SMD = 0.39,95% CI: 0.02, 0.77), SQFZ in 6 studies (SMD = 0.69,95%CI: 0.3, 1.07) (see Table 8). The rank probability analysis (Figure 8B) revealed the toptreatments:SQFZ (SUCRA: 83.74%). The funnel plot reflects whether there is potential bias (Figure 8C), the scatter points present an “inverted L-shaped” distribution (with dense clustering at the bottom and sparse distribution at the top of the vertical axis), indicating that small-sample studies dominate the negative effect results, suggesting the presence of bias.

**TABLE 8:** CD4+/CD8+ league table.

C	0.39 (0.02, 0.77)	0.35 (−0.56, 1.26)	0.44 (−0.49, 1.37)	0.39 (−0.29, 1.06)	0.29 (−0.18, 0.76)	0.69 (0.3, 1.07)
−0.39 (−0.77, −0.02)	FFKS	−0.04 (−1.03, 0.94)	0.05 (−0.96, 1.05)	−0.01 (−0.78, 0.76)	−0.1 (−0.71, 0.5)	0.29 (−0.25, 0.83)
−0.35 (−1.26, 0.56)	0.04 (−0.94, 1.03)	HQ	0.09 (−1.22, 1.39)	0.03 (−1.1, 1.16)	−0.06 (−1.08, 0.97)	0.34 (−0.66, 1.33)
−0.44 (−1.37, 0.49)	−0.05 (−1.05, 0.96)	−0.09 (−1.39, 1.22)	KA	−0.05 (−1.21, 1.1)	−0.15 (−1.19, 0.9)	0.25 (−0.77, 1.25)
−0.39 (−1.06, 0.29)	0.01 (−0.76, 0.78)	−0.03 (−1.16, 1.1)	0.05 (−1.1, 1.21)	KLT	−0.09 (−0.91, 0.73)	0.3 (−0.47, 1.08)
−0.29 (−0.76, 0.18)	0.1 (−0.5, 0.71)	0.06 (−0.97, 1.08)	0.15 (−0.9, 1.19)	0.09 (−0.73, 0.91)	SM	0.39 (−0.21, 1)
−0.69 (−1.07, −0.3)	−0.29 (−0.83, 0.25)	−0.34 (−1.33, 0.66)	−0.25 (−1.25, 0.77)	−0.3 (−1.08, 0.47)	−0.39 (−1, 0.21)	SQFZ

The ones marked in red are statistically significant.

#### 4.4.3 Tumor biomarker indicators

Tumor markers are biochemical substances produced either by neoplastic cells or by the host in response to malignancy and are detectable in blood, body fluids, or tissues (Zhou et al., 2024). They serve as adjuncts for early screening and diagnosis, facilitate the assessment of therapeutic efficacy and disease progression, enable surveillance for recurrence or metastasis, and guide prognostic evaluation as well as individualized treatment strategies (Zhou et al., 2024). A sustained decrease in tumor marker levels has been shown to correlate with attenuated malignant progression in breast cancer (Ni et al., 2024).

##### 4.4.3.1 Tumor biomarker indicators:CEA

**FIGURE 9:**
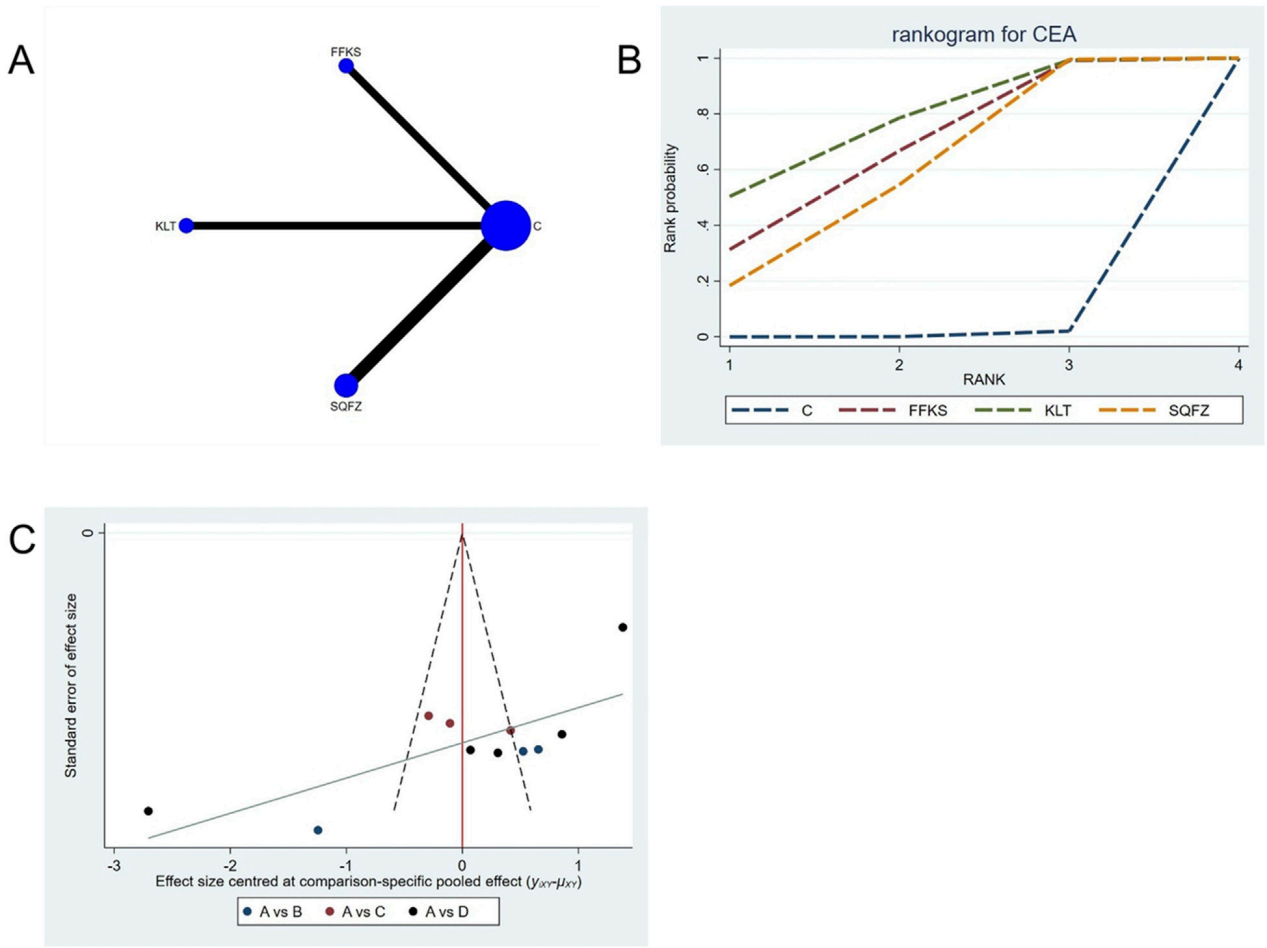
Network meta-analysis results for Efficacy. **(A)** Network plot **(B)** Cumulative probability ranking curve of different interventions. **(C)** Funnel plot. FFKS, Compound Sophora Injection; KLT, Kanglaite Injection; SQFZ, Shenqifuzheng Injection. T, Treatment; C, Control group.

Carcinoembryonic antigen (CEA), a broad-spectrum tumor marker in routine clinical practice (Gao et al., 2018), exhibits significant prognostic value in breast cancer and is commonly employed for therapeutic response evaluation, progression monitoring, and prognostic assessment in patients with metastatic breast cancer (Pestka, 1986). For this outcome measure, a total of 11 randomized controlled trials (RCTs)evaluating 3 Chinese herbal injections (CHIs) were included. The network structure of these interventions is presented in (Figure 9A). Among them, SQFZ combined with the conventional treatment group had the largest sample size and was investigated in the greatest number of studies compared to the control group. The deviance information criterion (DIC) difference was 0.01901. Pairwise comparisonsdemonstrated statistically significant superiority over the control group for the following treatments:SQFZ in 5 studies (SMD = −3.27, 95% CI: −5.56, −1.07), FFKS in 3 studies (SMD = −3.62, 95% CI: -6.51, −0.81),KLT in 3 studies (SMD = −4.1, 95% CI: −7.09, −1.04) (see Table 9). The rank probability analysis (Figure 9B) revealed the top treatments:KLT (SUCRA: 76.07%). The funnel plot reflects whether there is potential bias (Figure 9C), The asymmetry of the funnel plot suggests the potential presence of bias.

**TABLE 9:** CEA league table.

C	−3.62 (−6.51, −0.81)	−4.1 (−7.09, −1.04)	−3.27 (−5.56, −1.07)
3.62 (0.81, 6.51)	FFKS	−0.49 (−4.58, 3.75)	0.34 (−3.27, 4)
4.1 (1.04, 7.09)	0.49 (−3.75, 4.58)	KLT	0.83 (−3, 4.5)
3.27 (1.07, 5.56)	−0.34 (−4, 3.27)	−0.83 (−4.5, 3)	SQFZ

The ones marked in red are statistically significant.

##### 4.4.3.2 Tumor biomarker indicators:CA125

**FIGURE 10:**
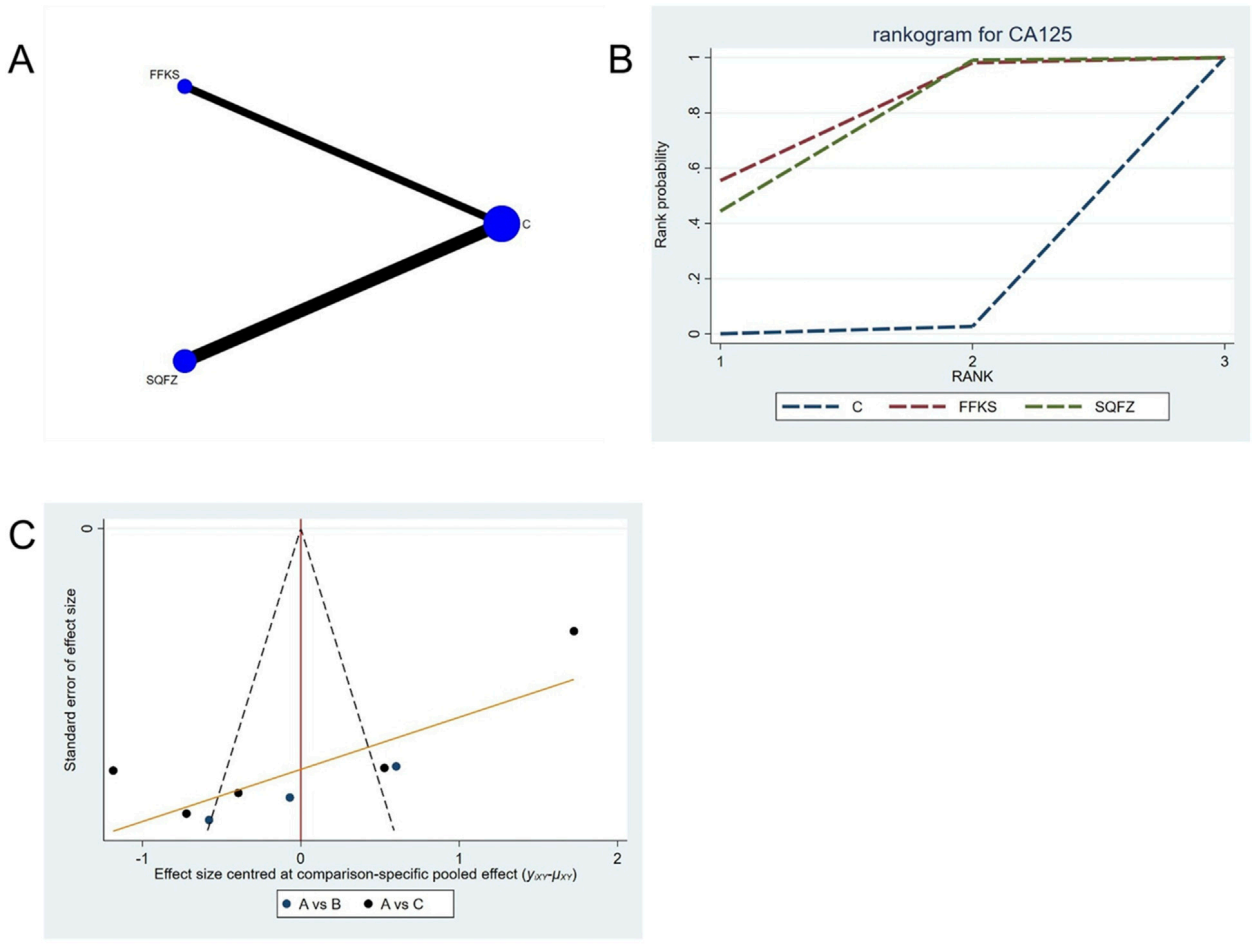
Network meta-analysis results for Efficacy. **(A)** Network plot **(B)** Cumulative probability ranking curve of different interventions. **(C)** Funnel plot. FFKS, Compound Sophora Injection; SQFZ, Shenqifuzheng Injection. T, Treatment; C, Control group.

CA125 is a high-molecular-weight glycoprotein synthesized by mesothelial cells of the peritoneum, pleura, pericardium, and endometrial epithelium (Kajiyama et al., 2017). Accumulating evidence indicates that elevated CA125 levels are closely associated with breast cancer pathogenesis and provide valuable prognostic information (Luo et al., 2022). For this outcome measure, a total of 8 randomized controlled trials (RCTs) evaluating 2 Chinese herbal injections (CHIs) were included. The network structure of these interventions is presented in (Figure 10A). Among them, SQFZ combined with the conventional treatment group had the largest sample size and was investigated in the greatest number of studies compared to the control group. The deviance information criterion (DIC) difference was 0.07047. Pairwise comparisons demonstrated statistically significant superiority over the control group for the following treatments: SQFZ in 5 studies (SMD = −8.27, 95% CI: −14.58, −1.94),FFKS in 3 studies (SMD = −8.89, 95% CI: −17.09, −0.75) (see Table 10). The rank probability analysis (Figure 10B) revealed the top treatments: FFKS (SUCRA:76.82%). The funnel plot reflects whether there is potential bias (Figure 10C), The scatter point shows a scarcity of data points on the right side (the positive effect area), with a left-skewed distribution, which demonstrates the presence of bias.

**TABLE 10:** CA125 league table.

C	−8.89 (−17.09, −0.75)	−8.27 (−14.58, −1.94)
8.89 (0.75, 17.09)	FFKS	0.61 (−9.72, 11.02)
8.27 (1.94, 14.58)	−0.61 (−11.02, 9.72)	SQFZ

The ones marked in red are statistically significant.

##### 4.4.3.3 Tumor biomarker indicators:CA153

**FIGURE 11:**
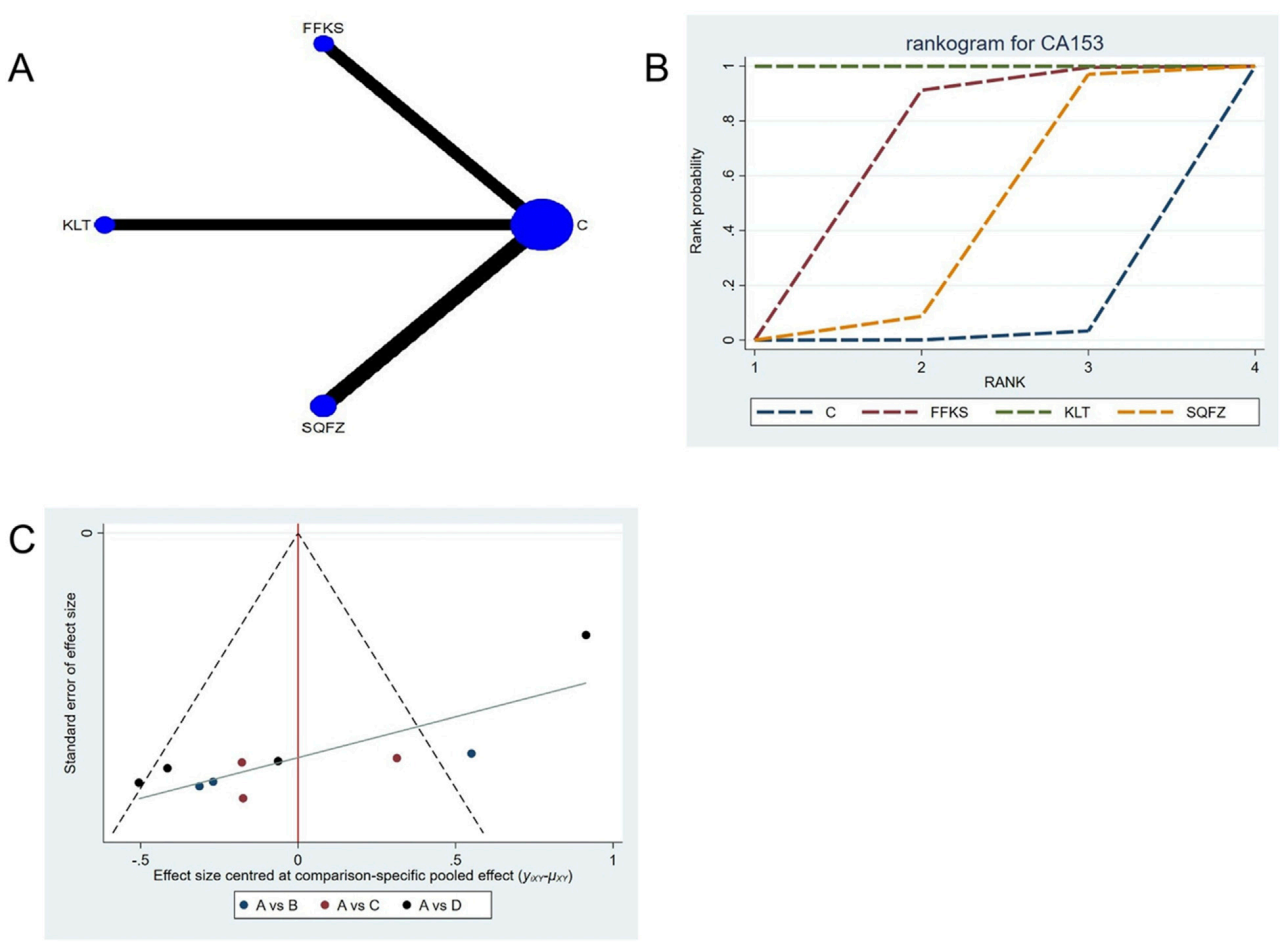
Network meta-analysis results for Efficacy. **(A)** Network plot **(B)** Cumulative probability ranking curve of different interventions. **(C)** Funnel plot. FFKS, Compound Sophora Injection; KLT, Kanglaite Injection; SQFZ, Shenqifuzheng Injection. T, Treatment; C, Control group.

CA153 (carbohydrate antigen 153) is among the most widely utilized serum tumor markers for breast cancer (Fang et al., 2017); however, its clinical utility is limited in advanced-stage disease, and its diagnostic sensitivity and specificity are comparatively low in early-stage cases. Consequently, CA15-3 is best employed as an adjunctive observational indicator rather than a standalone diagnostic tool (Liu et al., 2018). For this outcome measure, a total of 10 randomized controlled trials (RCTs) evaluating 3 Chinese herbal injections (CHIs) were included. The network structure of these interventions is presented in (Figure 11A). Among them, SQFZ combined with the conventional treatment group had the largest sample size and was investigated in the greatest number of studies compared to the control group. The deviance information criterion (DIC) difference was 0.04578. Pairwise comparisons demonstrated statistically significant superiority over the control group for the following treatments (see Table 11): FFKS in 3 studies (SMD = −8.87, 95% CI: −14.48, −3.39), KLT in 3 studies (SMD = −62.12, 95% CI: −70.94, −53.37). The rank probability analysis (Figure 11B) revealed the top three treatments:KLT (SUCRA: 99.99%), FFKS (SUCRA: 63.63%), SQFZ (SUCRA: 35.25%). The funnel plot reflects whether there is potential bias (Figure 11C), the substantial asymmetry of the scatter plot indicates the presence of significant bias.

**TABLE 11:** CA153 league table.

C	−8.87 (−14.48, −3.39)	−62.12 (−70.94, −53.37)	−4.43 (−9.24, 0.27)
8.87 (3.39, 14.48)	FFKS	−53.24 (−63.56, −42.86)	4.45 (−2.91, 11.78)
62.12 (53.37, 70.94)	53.24 (42.86, 63.56)	KLT	57.68 (47.71, 67.65)
4.43 (−0.27, 9.24)	−4.45 (−11.78, 2.91)	−57.68 (−67.65, −47.71)	SQFZ

The ones marked in red are statistically significant.

#### 4.4.4 Adverse reactions

Adverse reactions were assessed by changes in WBC, PLT, and HGB levels pre- and post-treatment, as described below.

##### 4.4.4.1 Adverse reaction: WBC count

**FIGURE 12:**
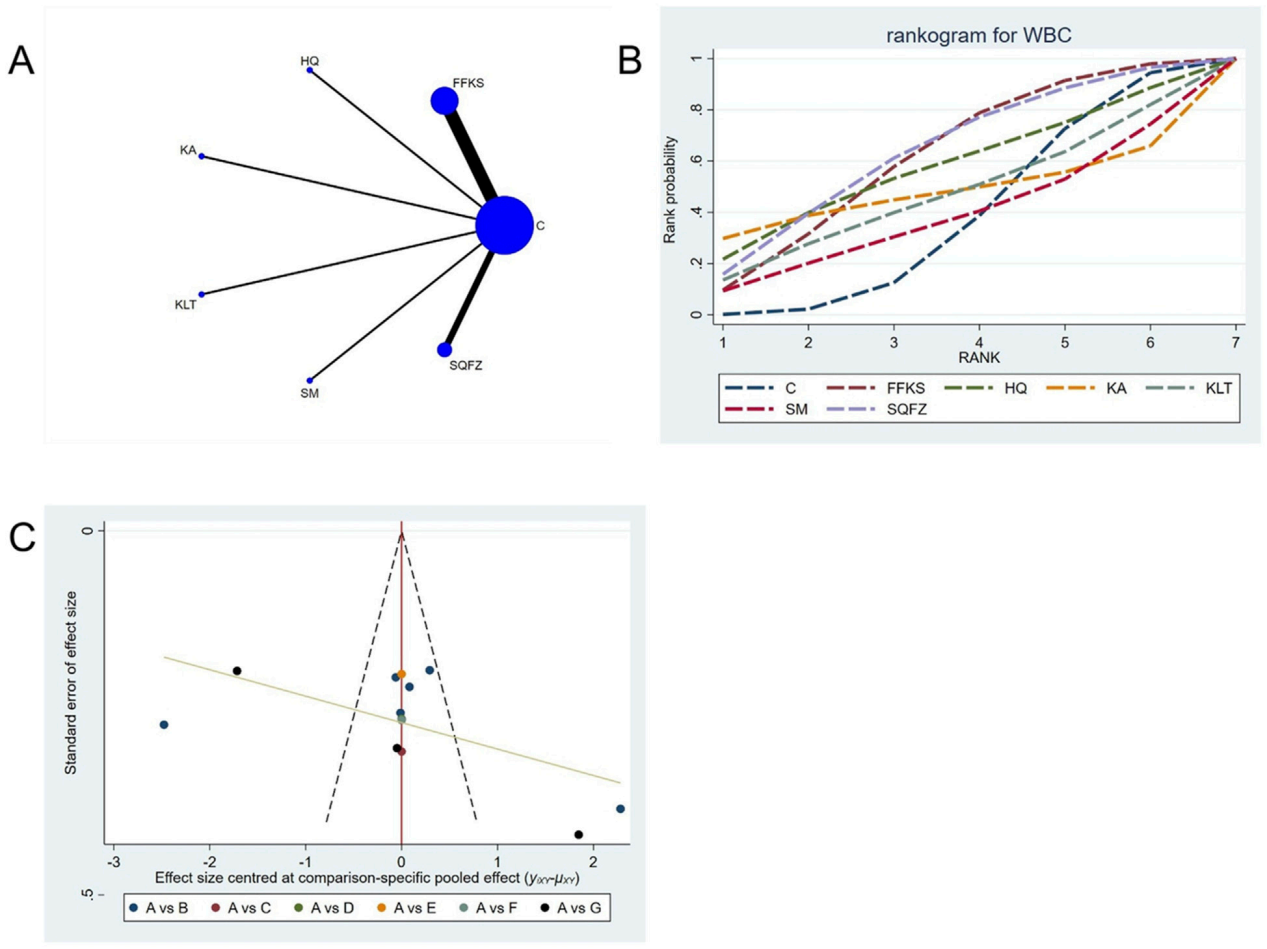
Network meta-analysis results for Efficacy. **(A)** Network plot **(B)** Cumulative probability ranking curve of different interventions. **(C)** Funnel plot. FFKS, Compound Sophora Injection; HQ, Huangqi Injection; KA, Kangai Injection; KLT, Kanglaite Injection; SM, Shenmai Injection; SQFZ, Shenqifuzheng Injection. T, Treatment; C, Control group.

For this outcome measure, a total of 13 randomized controlled trials (RCTs) evaluating 6 Chinese herbal injections (CHIs) were included. The network structure of these interventions is presented in (Figure 12A). Among them, the FFKS combined with the conventional treatment group had the largest sample size and was investigated in the greatest number of studies compared to the control group. The deviance information criterion (DIC) difference was 0.04475. Pairwise comparisons demonstrated no statistically significant differences compared to the control group (see Table 12). The rank probability analysis (Figure 12B) revealed the top treatments:SQFZ (SUCRA: 63.15%). The funnel plot reflects whether there is potential bias (Figure 12C), the scatter point is essentially symmetrically distributed (with scatter points of similar density above and below the center at SMD = 0), suggesting a low risk of publication bias for this indicator.

**TABLE 12:** WBC league table.

C	1.15 (−1.37, 3.64)	1.13 (−4.92, 7.16)	0.2 (−11.55, 11.96)	0.27 (−5.71, 6.28)	−0.39 (−6.45, 5.64)	1.35 (−2.13, 4.83)
−1.15 (−3.64, 1.37)	FFKS	−0.02 (−6.59, 6.51)	−0.95 (−12.96, 11.06)	−0.87 (−7.38, 5.62)	−1.53 (−8.07, 5.02)	0.21 (−4.08, 4.49)
−1.13 (−7.16, 4.92)	0.02 (−6.51, 6.59)	HQ	−0.94 (−14.1, 12.29)	−0.84 (−9.36, 7.65)	−1.5 (−10.11, 7.02)	0.23 (−6.76, 7.2)
−0.2 (−11.96, 11.55)	0.95 (−11.06, 12.96)	0.94 (−12.29, 14.1)	KA	0.11 (−13.14, 13.23)	−0.58 (−13.77, 12.61)	1.16 (−11.11, 13.42)
−0.27 (−6.28, 5.71)	0.87 (−5.62, 7.38)	0.84 (−7.65, 9.36)	−0.11 (−13.23, 13.14)	KLT	−0.67 (−9.17, 7.87)	1.07 (−5.91, 8.03)
0.39 (−5.64, 6.45)	1.53 (−5.02, 8.07)	1.5 (−7.02, 10.11)	0.58 (−12.61, 13.77)	0.67 (−7.87, 9.17)	SM	1.74 (−5.27, 8.71)
−1.35 (−4.83, 2.13)	−0.21 (−4.49, 4.08)	−0.23 (−7.2, 6.76)	−1.16 (−13.42, 11.11)	−1.07 (−8.03, 5.91)	−1.74 (−8.71, 5.27)	SQFZ

The ones marked in red are statistically significant.

##### 4.4.4.2 Adverse reaction: PLT count

**FIGURE 13:**
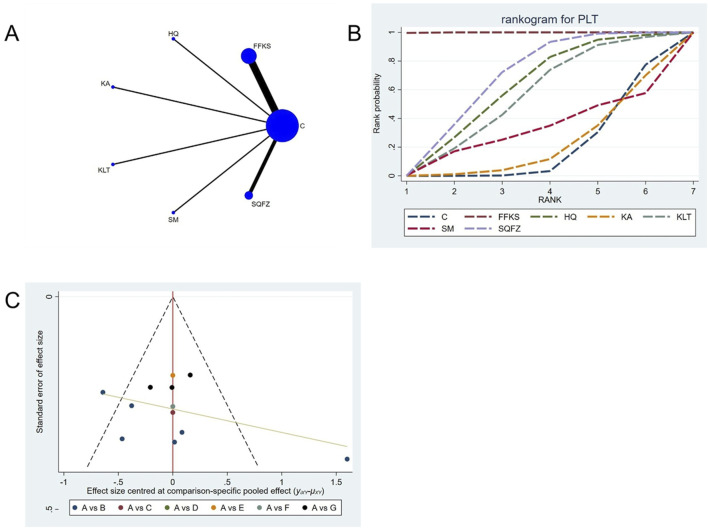
Network meta-analysis results for Efficacy. **(A)** Network plot **(B)** Cumulative probability ranking curve of different interventions. **(C)** Funnel plot. FFKS, Compound Sophora Injection; HQ, Huangqi Injection; KA, Kangai Injection; KLT, Kanglaite Injection; SM, Shenmai Injection; SQFZ, Shenqifuzheng Injection. T, Treatment; C, Control group.

For this outcome measure, a total of 13 randomized controlled trials (RCTs) evaluating 6 Chinese herbal injections (CHIs) were included. The network structure of these interventions is presented in (Figure 13A). Among them, FFKS combined with the conventional treatment group had the largest sample size and was investigated in the greatest number of studies compared to the control group. The deviance information criterion (DIC) difference was −0.11165. Pairwise comparisons demonstrated statistically significant superiority over the control group for the following treatments (see Table 13): FFKS in 6 studies (SMD = 49.37, 95% CI: 43.23, 55.69), SQFZ in 3 studies (SMD = 13.78, 95% CI: 4.69, 22.75). The rank probability analysis (Figure 13B) revealed the top treatments: FFKS (SUCRA: 99.93%). The funnel plot reflects whether there is potential bias (Figure 13C), the scatter point shows a right-skewed distribution, suggesting the presence of potential bias.

**TABLE 13:** PLT league table.

C	49.37 (43.23, 55.69)	11.98 (−2.62, 26.61)	−0.06 (−13.54, 13.4)	10.19 (−5.2, 25.49)	0.82 (−32, 33.91)	13.78 (4.69, 22.75)
−49.37 (−55.69, −43.23)	FFKS	−37.34 (−53.37, −21.53)	−49.4 (−64.41, −34.75)	−39.16 (−55.86, −22.79)	−48.55 (−81.98, −14.89)	−35.59 (−46.8, −24.82)
−11.98 (−26.61, 2.62)	37.34 (21.53, 53.37)	HQ	−12.04 (−31.9, 7.74)	−1.81 (−23.09, 19.29)	−11.15 (−47.16, 25)	1.79 (−15.55, 18.89)
0.06 (−13.4, 13.54)	49.4 (34.75, 64.41)	12.04 (−7.74, 31.9)	KA	10.25 (−10.15, 30.52)	0.96 (−34.31, 36.31)	13.84 (−2.48, 29.91)
−10.19 (−25.49, 5.2)	39.16 (22.79, 55.86)	1.81 (−19.29, 23.09)	−10.25 (−30.52, 10.15)	KLT	−9.33 (−45.58, 27.08)	3.57 (−14.26, 21.33)
−0.82 (−33.91, 32)	48.55 (14.89, 81.98)	11.15 (−25, 47.16)	−0.96 (−36.31, 34.31)	9.33 (−27.08, 45.58)	SM	12.94 (−21.23, 47.04)
−13.78 (−22.75, −4.69)	35.59 (24.82, 46.8)	−1.79 (−18.89, 15.55)	−13.84 (−29.91, 2.48)	−3.57 (−21.33, 14.26)	−12.94 (−47.04, 21.23)	SQFZ

The ones marked in red are statistically significant.

##### 4.4.4.3 Adverse reaction: HGB count

**FIGURE 14:**
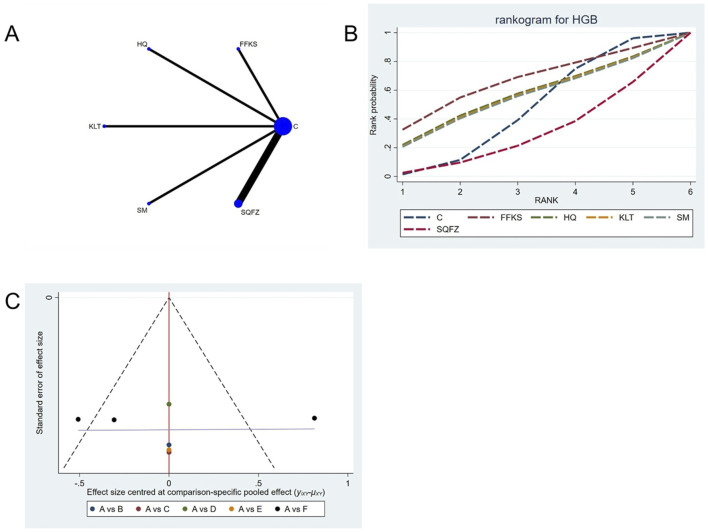
Network meta-analysis results for Efficacy. **(A)** Network plot **(B)** Cumulative probability ranking curve of different interventions. **(C)** Funnel plot. FFKS, Compound Sophora Injection; HQ, Huangqi Injection; KLT, Kanglaite Injection; SM, Shenmai Injection; SQFZ, Shenqifuzheng Injection. T, Treatment; C, Control group.

For this outcome measure, a total of 7 randomized controlled trials (RCTs) evaluating 5 Chinese herbal injections (CHIs) were included. The network structure of these interventions is presented in (Figure 14A). Among them, the SQFZ combined with the conventional treatment group had the largest sample size and was investigated in the greatest number of studies compared to the control group. The deviance information criterion (DIC) difference was 0.04621. Pairwise comparisons demonstrated no statistically significant differences compared to the control group (see Table 14). The rank probability analysis (Figure 14B) revealed the top treatments: FFKS(SUCRA: 65.04%). The funnel plot reflects whether there is potential bias (Figure 14C), the scatter point is evenly distributed around the center at SMD = 0, with no obvious shift, suggesting a low risk of publication bias.

**TABLE 14:** HGB league table.

C	30.8 (−93.79, 155.49)	14.85 (−109.12, 138.45)	13.4 (−110.81, 137.3)	12.49 (−112.48, 136.37)	−20.33 (−91.97, 51.34)
−30.8 (−155.49, 93.79)	FFKS	−15.83 (−192.22, 159.32)	−17.22 (−192.95, 158.38)	−18.3 (−194.55, 158.09)	−51.01 (−194.93, 92.84)
−14.85 (−138.45, 109.12)	15.83 (−159.32, 192.22)	HQ	−1.53 (−176.01, 174.03)	−2.25 (−178.13, 173.91)	−34.99 (−179.06, 107.81)
−13.4 (−137.3, 110.81)	17.22 (−158.38, 192.95)	1.53 (−174.03, 176.01)	KLT	−1.01 (−176.4, 174.63)	−33.62 (−176.77, 109.28)
−12.49 (−136.37, 112.48)	18.3 (−158.09, 194.55)	2.25 (−173.91, 178.13)	1.01 (−174.63, 176.4)	SM	−32.67 (−176.35, 110.77)
20.33 (−51.34, 91.97)	51.01 (−92.84, 194.93)	34.99 (−107.81, 179.06)	33.62 (−109.28, 176.77)	32.67 (−110.77, 176.35)	SQFZ

The ones marked in red are statistically significant.

### 4.5 Sensitivity analysis and publication bias

Funnel plots were used to assess publication bias across all outcome measures. Points in different colors represent comparisons between Chinese herbal injections (CHIs) and conventional therapy, as shown in Figures 3–14. The slopes of the fitted straight lines of the funnel plots of HGB were all close to the center line, showing a bilaterally symmetric distribution, indicating that there was no publication bias. However, asymmetrical distributions were observed in funnel plots for the following outcomes: Efficacy, Karnofsky Performance Status (KPS), CD3^+^, CD4^+^, CD8^+^, CD4+/CD8+ ratio,CEA, CA125, CA153, white blood cell count (WBC), and platelet count (PLT). This asymmetry indicates potential publication bias (see Figures 3–14).

### 4.6 Assessment of transitivity and consistency

#### 4.6.1 Assessment of transitivity

1. Baseline Characteristics

As shown in Table 2, all patients included in the studies were Chinese women with breast cancer. Although the age range was broad (20–81 years), the mean ages across study groups were comparable. It should be noted that some studies had inconsistent reporting of TNM staging, which may affect transitivity to some extent. However, this inconsistency is unlikely to lead to systematic bias overall.

2. Methodological Homogeneity

All studies were conducted in China and were open-label, introducing a consistent direction of performance bias.

3. Network Structure

All networks were star-shaped, with all interventions compared to chemotherapy alone. This structure supports the assumption of transitivity.

4. Clinical Homogeneity of Interventions

All studies evaluated Chinese Herbal Injections (CHI) as adjuncts to chemotherapy. However, there were differences in the specific chemotherapy regimens used across studies (e.g., TAC, AC, EC-T). These differences may pose a potential threat to transitivity. Nevertheless, since these regimens are all commonly used in clinical practice, the overall treatment framework remains comparable.

In summary, based on the cumulative evidence—particularly the critical imbalance in chemotherapy regimens (clinical homogeneity)—the transitivity assumption may not hold for some outcomes. Therefore, the results of indirect comparisons should be interpreted with caution.

#### 4.6.2 Assessment of consistency

Given the star-shaped network structure (no closed loops), local inconsistency could not be assessed via node-splitting. Therefore, global inconsistency was evaluated by comparing the Deviance Information Criterion (DIC) between consistency and inconsistency models. For all outcomes, the difference in DIC was below 5 (maximum difference: 0.11962 for CD3^+^), indicating no significant global inconsistency.

## 5 Discussion

To our knowledge, this represents the first network meta-analysis (NMA) in the past 5 years to systematically evaluate the efficacy and safety profiles of various Chinese herbal injections (CHIs) combined with chemotherapy for breast cancer treatment. This comprehensive NMA incorporated the most recent data from 44 eligible randomized controlled trials (RCTs).

Our results demonstrate that SQFZ combined with chemotherapy is the most effective intervention for improving treatment efficacy, quality of life, post-chemotherapy CD4+/CD8+levels, and inhibiting post-chemotherapy WBC reduction. FFKS combined with chemotherapy proved most effective for improving post-chemotherapy CD4^+^ and CD8^+^ levels, lowering tumor marker CA125, and suppressing declines in PLT and hemoglobin HGB levels. KA combined with chemotherapy is the most effective intervention for improving post-chemotherapy CD3^+^ levels. KLT combined with chemotherapy is the most effective intervention for reducing tumor markers CEA and CA125 levels.

As the most recommended traditional Chinese medicine injection for breast cancer patients undergoing combination chemotherapy in this network meta-analysis,SQFZ has significant advantages in enhancing the efficacy of chemotherapy, improving the quality of life of breast cancer patients, ameliorating the CD4+/CD8+ T-cell ratio, and inhibiting the decrease in WBC count after chemotherapy. SQFZ is composed of extracts from Astragalus membranaceus and Codonopsis pilosula (Jia et al., 2019; Wu et al., 2021), both of which are traditional Chinese medicines (TCM) with “energy-boosting and body-strengthening” effects (Luo et al., 2023). SQFZ is a traditional Chinese medicine preparation composed of Astragaloside IV, Astragalus polysaccharides, Codonopsis polysaccharides, and other active components. Astragaloside IV, a major active compound, exhibits significant anticancer effects by downregulating the expression of STAT3 and NF-κB in tumor cells, thereby inhibiting the secretion of IL-10 (Kong et al., 2024; Meng et al., 2020). Both Astragalus polysaccharides and Codonopsis polysaccharides possess antitumor and immunomodulatory properties (Han et al., 2025; Zheng et al., 2024). Astragalus polysaccharides, in particular, demonstrate robust immune-enhancing characteristics, stimulating the proliferation of B cells and cytokine production in mice, which may be associated with the improvement of the CD4+/CD8+ T-cell ratio (Chen et al., 2019). Additionally, SQFZ has been proven to counteract the side effects of chemotherapeutic drugs, such as significantly alleviating myelosuppression in cancer patients. This may be related to inhibiting the decrease in WBC count after chemotherapy. Collectively, SQFZ not only achieves maximal antitumor efficacy in breast cancer therapy but also most markedly improves the quality of life of patients undergoing chemotherapy, while exerting favorable effects on both immune function and leukocyte counts. It should therefore be regarded as the preferred traditional Chinese medicine injection for combined chemotherapy in the clinical management of breast cancer, demonstrating significant therapeutic value.

FFKS, a dual-herb formulation derived from Radix Sophorae Flavescentis and Rhizoma Heterosmilacis Yunnanensis (Gao et al., 2021; Zhou et al., 2020), demonstrates significant efficacy in improving post-chemotherapy CD4^+^ and CD8^+^ levels, reducing tumor marker CA125 levels, and suppressing declines in platelet (PLT) and hemoglobin (HGB) levels. The primary pharmacologically active constituents responsible for the anticancer effects of FFKS are matrine and oxymatrine (Yu et al., 2023). Matrine induces cell cycle arrest at the G0/G1 phase and mitochondrial apoptosis in malignant cells, while concurrently downregulating the expression of Ki-67 and Survivin, thereby reducing serum CA125 concentration (Wu et al., 2023). Oxymatrine inhibits the proliferation of breast cancer cells (MCF-7, MDA-MB-231) in a time- and concentration-dependent manner. It exerts its anti-breast cancer activity by inhibiting the PI3K/Akt signaling pathway through the activation of PI3K and Akt dephosphorylation (Guo et al., 2022). Furthermore, FFKS also mitigates tumor-associated macrophage (TAM)-mediated immunosuppression by suppressing the tumor necrosis factor receptor 1 (TNFR1) signaling pathway, which is likely a key mechanism underlying the observed restoration of CD4^+^ and CD8^+^ T cell populations following chemotherapy (Yang et al., 2020). Additional studies have confirmed the significant efficacy of FFKS in alleviating chemotherapy-induced myelosuppression, corroborating its effect in inhibiting the decline of platelets (PLT) and hemoglobin (HGB) (Fang et al., 2025; Hu et al., 2025). Collectively, these findings indicate that FFKS primarily functions to enhance immune competence and attenuate chemotherapy-related toxicities, rendering it a rational adjunct for patients experiencing post-chemotherapy immunodeficiency and myelosuppression.

KA combined with chemotherapy was the most effective intervention for enhancing post-chemotherapy CD3^+^ levels. KA, a standardized Chinese herbal antitumor formulation composed of oxymatrine (OMT) (Chen et al., 2023; Chen et al., 2021; Wang et al., 2020), Panax ginseng C.A.Mey., and Astragalus membranaceus (Fisch.), is clinically recognized for its dual “Qi-replenishing and Zheng-strengthening” therapeutic properties (Guo et al., 2024). The primary active constituents responsible for the anticancer effects of KA are ginsenosides, matrine, and oxymatrine (Luo et al., 2020). The antitumor effects of matrine and oxymatrine have been previously described and will not be reiterated here. Regarding the remaining component, ginsenosides modulate immune function by enhancing immune responses to eliminate tumor cells (Li et al., 2021), This mechanism corroborates the observed elevation in CD3^+^ T cells associated with KA administration in the current study. It is worth noting that, among the immune function indicators, CD3^+^ has unique characteristics compared with CD4^+^, CD8^+^, and the CD4+/CD8+ratio. CD3^+^ is a hallmark molecule on the surface of T cells, and almost all mature T cells express CD3^+^. A decrease in CD3^+^ levels typically indicates a reduction in the total number of T cells, which reflects the extensive immunosuppressive effects of chemotherapy and is both widespread and typical (Cai et al., 2022; Sun et al., 2018). In conclusion, in clinical practice, if a decline in immune function indicators characterized by a reduction in CD3^+^ is observed following breast cancer chemotherapy, the adjunctive use of KA should be considered.

KLT combined with chemotherapy was the most effective intervention for reducing CEA and CA153 levels. KLT, a broad-spectrum antitumor agent extracted from coix seed, is widely used in cancer treatment (Huang et al., 2020; Shao et al., 2025). The pharmacologically active constituents of KLT include Coixenolide (the oil of Coix lacryma-jobi) (Wang et al., 2021), glycerol, and injectable glycerin, among which Coixenolide serves as the primary component responsible for its anticancer effects. Coixenolide reportedly induces apoptosis in various tumor cells through multiple pathways, such as inhibiting tumor cell mitosis at the G2/M phase or stimulating immune functions, thereby reducing tumor markers (Cheng et al., 2014; Jiang et al., 2024). Therefore, in clinical practice, KLT can be considered as an adjunctive therapy for patients with persistently elevated CEA and CA153 levels. However, given the significant bias associated with CA153, the results should also be interpreted with caution.

In the end, SQFZ represents the most significant traditional Chinese medicine injection for breast cancer treatment identified in this study, demonstrating dual benefits of therapeutic efficacy and improved quality of life. Its evaluation across a substantial patient population underscores both its broad applicability and marked clinical significance, establishing it as the most important finding in this article.

### 5.1 Limitations

The current network meta-analysis (NMA) has several unavoidable limitations. First, While most outcomes showed consistent treatment effects, funnel plot asymmetry was observed for specific biomarkers (Figures 3–5, 7–9, 11). This may reflect:Selective publication of smaller studies with positive results (e.g., KLT for CA153), potentially inflating effect estimates. Heterogeneity in measurement protocols for tumor markers (CEA/CA153), as indicated by scattered distributions. Notably, safety outcomes (HGB: Figure 14C; WBC: Figure 12C) showed better symmetry, supporting the robustness of myelosuppression-related conclusions. Second, the relatively small number of partial intervention studies included in this NMA may have influenced the conclusions. although randomized controlled trials were included, the lack of blinding in some studies might have introduced bias. Third, although randomized controlled trials were included, the lack of blinding might have introduced bias. Fourth, limitations in data extracted from the included studies precluded more detailed subgroup analyses, potentially affecting the final results. Therefore, we recommend: 1 Prioritize large samples (>200/arm) to mitigate small-study effects. 2 implementing prospective registration of RCTs to ensure timeline transparency and enhance methodological quality; 3 conducting RCTs in strict compliance with updated clinical diagnosis and treatment guidelines; 4 prioritizing long-term and clinically significant endpoints in RCTs involving cancer patients. Given these limitations, more rigorously designed, high-quality RCTs are required to confirm the therapeutic efficacy of Chinese herbal injections (CHIs) combined with chemotherapy in breast cancer patients. Furthermore, the transitivity assumption may be compromised by heterogeneity in chemotherapy regimens. Although no global inconsistency was detected, the star-shaped network precluded a full evaluation of local inconsistency, which should be considered when interpreting the results.

## 6 Conclusion

In summary, current evidence demonstrates that Chinese herbal injection (CHI)-chemotherapy combinations confer greater clinical benefits for breast cancer (BC) patients compared to conventional chemotherapy alone. Among eight therapeutic regimens evaluated, four interventions exhibited superior efficacy profiles: SQFZ combined with chemotherapy, FFKS combined with chemotherapy, KA combined with chemotherapy, and KLT combined with chemotherapy. Especially, SQFZ combined with chemotherapy showed the best results in the two main outcome indicators of efficacy and quality of life, and it was also the best in improving CD4+/CD8+ levels and inhibiting WBC reduction after chemotherapy. It can guide the selection of Chinese medicine injections in breast cancer treatment. However, limitations in the number and quality of included studies, along with potential bias, necessitate confirmation through further high-quality, large-scale, double-blind RCTs.

## Data Availability

The original contributions presented in the study are included in the article/Supplementary Material, further inquiries can be directed to the corresponding author.
