# Supplementary material for: 8 specific Chinese herbal injections combined with chemotherapy for breast cancer: a systematic review and network meta-analysis of comparative safety and efficacy
Source: Front Pharmacol. 2025 Oct 3;16:1661803. doi: 10.3389/fphar.2025.1661803 (PMC12531140; doi:10.3389/fphar.2025.1661803)
Supplement: Supplementary file 5 [file Supplementaryfile2.docx]

**Appendix 2** (The search process of PubMed and Web of Science is not included in the file uploaded separately). The PubMed search results are in Appendix 5. The search records of Web of Science are in Appendix 6.

CNKI (1393)

中药 OR 注射 OR 华蟾素 OR 消癌平 OR 艾迪 OR 苦参 OR 黄芪 OR 参芪 OR 康莱特

乳腺癌 OR 乳腺肿瘤 OR 乳腺癌症 OR 人类乳腺肿瘤 OR 乳房癌 OR 乳癌 OR 乳房肿瘤 OR 乳岩

随机

（主题：中药 + 注射 + 华蟾素 + 消癌平 + 艾迪 + 苦参 + 黄芪 + 参芪 + 康莱特） AND （主题：乳腺癌 + 乳腺肿瘤 ＋ 乳腺癌症 + 人类乳腺肿瘤 + 乳房癌 + 乳癌 + 乳房肿瘤 + 乳岩） AND （篇关摘：随机（精确））

Cochrane Library 1117

ID Search Hits

#1 MeSH descriptor: [Medicine, Chinese Traditional] explode all trees 1792

#2 (Chinese medicine):ti,ab,kw OR (Chung I Hsueh):ti,ab,kw OR (Traditional Tongue Assessment*):ti,ab,kw OR (Traditional Tongue Diagnos*):ti,ab,kw OR (Zhong Yi Xue):ti,ab,kw 17011

#3 (injection*):ti,ab,kw 120692

#4 MeSH descriptor: [Breast Neoplasms] explode all trees 20444

#5 (Breast*Cancer):ti,ab,kw OR (Breast Carcinoma*):ti,ab,kw OR (breast Malignanc*):ti,ab,kw OR (Breast Neoplasm*):ti,ab,kw OR (Breast Tumor*):ti,ab,kw 32672

#6 (Ca breast):ti,ab,kw OR (Cancer*,Mammary):ti,ab,kw OR (Carcinoma*,Human Mammary):ti,ab,kw OR (Malignant Tumor of Breast):ti,ab,kw OR (Mammary Malignanc*):ti,ab,kw 1578

#7 (random*):ti,ab,kw 1368045

#8 (#1 OR #2 OR #3) AND (#4 OR #5 OR #6) AND (#7) 1129

Embase1717

Session Results

.......................................................

No. Query Results Results Date

#5. (#1 OR #2) AND (#3 OR #4) 1,717 10 Sep 2024

#4. 'chinese herbal medicine':ab,ti OR 'chinese 62,484 10 Sep 2024

medicine':ab,ti OR 'chinese traditional

medicine':ab,ti OR 'chung i hsueh':ab,ti OR

'traditional chinese medicine':ab,ti OR

'traditional tongue assessment?':ab,ti OR

'traditional tongue diagnos?s':ab,ti OR 'zhong yi

xue':ab,ti

#3. 'chinese herbal medicine'/exp 82,972 10 Sep 2024

#2. 'breast*cancer':ab,ti OR 'breast 82,025 10 Sep 2024

carcinoma*':ab,ti OR 'breast malignanc*':ab,ti OR

'breast neoplasm*':ab,ti OR 'breast tumor*':ab,ti

OR 'ca breast':ab,ti OR 'carcinoma*,human

mammary':ab,ti OR 'malignant tumor of

breast':ab,ti OR 'mammary malignanc*':ab,ti

#1. 'breast cancer'/exp 620,593 10 Sep 2024

PUBMED523

See appendix5.exc

sinimed2167

序号 检索表达式 命中文献数 检索时间

1) "中药"[常用字段:智能] OR "注射"[常用字段:智能] OR "华蟾素"[常用字段:智能] OR "消癌平"[常用字段:智能] OR "艾迪"[常用字段:智能] OR "苦参"[常用字段:智能] OR "黄芪"[常用字段:智能] OR "参芪"[常用字段:智能] OR "康莱特"[常用字段:智能] 1133333 2024-09-14 09:45:49.0

2) "乳腺癌"[常用字段:智能] OR "乳腺肿瘤"[常用字段:智能] OR "乳腺癌症"[常用字段:智能] OR "人类乳腺肿瘤"[常用字段:智能] OR "乳房癌"[常用字段:智能] OR "乳癌"[常用字段:智能] OR "乳房肿瘤"[常用字段:智能] OR "乳岩"[常用字段:智能] 140200 2024-09-14 09:48:20.0

3) "随机"[常用字段:智能] 1987744 2024-09-14 09:49:08.0

4) "中药"[不加权:扩展] 114983 2024-09-14 09:51:58.0

5) "乳腺肿瘤"[不加权:扩展] 117370 2024-09-14 09:52:28.0

6) ((#4) OR (#1)) 1133333 2024-09-14 09:54:11.0

7) ((#5) OR (#2)) 140200 2024-09-14 09:55:00.0

8) (#7) AND (#6) AND (#3) 2167 2024-09-14 09:56:19.0

VIP Information832

题名或关键词=中药 + 注射 + 华蟾素 + 消癌平 + 艾迪 + 苦参 + 黄芪 + 参芪 + 康莱特 AND 题名或关键词=乳腺癌 + 乳腺肿瘤 ＋ 乳腺癌症 + 人类乳腺肿瘤 + 乳房癌 + 乳癌 + 乳房肿瘤 + 乳岩 AND 摘要=随机

Wanfang874

题名或关键词:(中药 OR 注射 OR 华蟾素 OR 消癌平 OR 艾迪 OR 苦参 OR 黄芪 OR 参芪 OR 康莱特) and 题名或关键词:(乳腺癌 OR 乳腺肿瘤 OR 乳腺癌症 OR 人类乳腺肿瘤 OR 乳房癌 OR 乳癌 OR 乳房肿瘤 OR 乳岩) and 摘要:(随机)

web of science 1173

See appendix5
