# Supplementary material for: 8 specific Chinese herbal injections combined with chemotherapy for breast cancer: a systematic review and network meta-analysis of comparative safety and efficacy
Source: Front Pharmacol. 2025 Oct 3;16:1661803. doi: 10.3389/fphar.2025.1661803 (PMC12531140; doi:10.3389/fphar.2025.1661803)
Supplement: Supplementary file 6 [file Supplementaryfile4.docx]

Appendix 4R script Continuity

The table header must be consistent with the following.


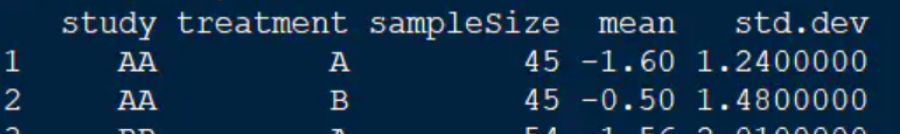


1.chooseCRANmirror()

2.install.packages("gemtc")

3.library(gemtc)

library(coda)

4.setwd("D:\\工作目录")

5.data<-read.csv("D:\\工作目录\\HADS.csv")

6.data

7.network <- mtc.network(data)

8.plot(network)

9.model<-mtc.model(network,type="consistency",n.chain=4,likelihood="normal",link="identity",linearModel="random")

10.results <- mtc.run(model, n.adapt = 20000, n.iter = 50000, thin = 1)

11.summary(results)

12.modelume<-mtc.model(network,type="ume",n.chain=4,likelihood="normal",link="identity",linearModel="random")

13.resultsume <- mtc.run(modelume, n.adapt = 20000, n.iter = 50000, thin = 1)

14.summary(resultsume)

Under the consistency model and the inconsistency model, if the difference in the DIC results is less than 5, it indicates that the consistency is good and the process can continue.

15.ranks <- rank.probability(results)

Or ranks <- rank.probability(results,preferredDirection=-1)

16.print(ranks)

17.write.csv(ranks, "rank.csv")

18.plot(ranks, beside = TRUE)

19.sucra(ranks)

20.tb<-relative.effect.table(results)

21.tb1<-round(tb,2)

22.write.csv(tb1, "结局1.csv")
